# Protocols for Understanding the Redox Behavior of Copper-Containing Systems

**DOI:** 10.1021/acsomega.2c05484

**Published:** 2022-11-30

**Authors:** Thomas Malcomson, Peter Repiščák, Stefan Erhardt, Martin J. Paterson

**Affiliations:** † Department of Chemistry, School of Natural Sciences, The University of Manchester, ManchesterM13 9PL, U.K.; ‡ Beatson Institute for Cancer Research, University of Glasgow, Garscube Estate Switchback Road, BearsdenG61 1QH, U.K.; § School of Applied Sciences, Edinburgh Napier University, EdinburghEH11 4BN, Scotland, U.K.; ∥ Institute of Chemical Sciences, School of Engineering and Physical Sciences, Heriot-Watt University, EdinburghEH14 4AS, U.K.

## Abstract

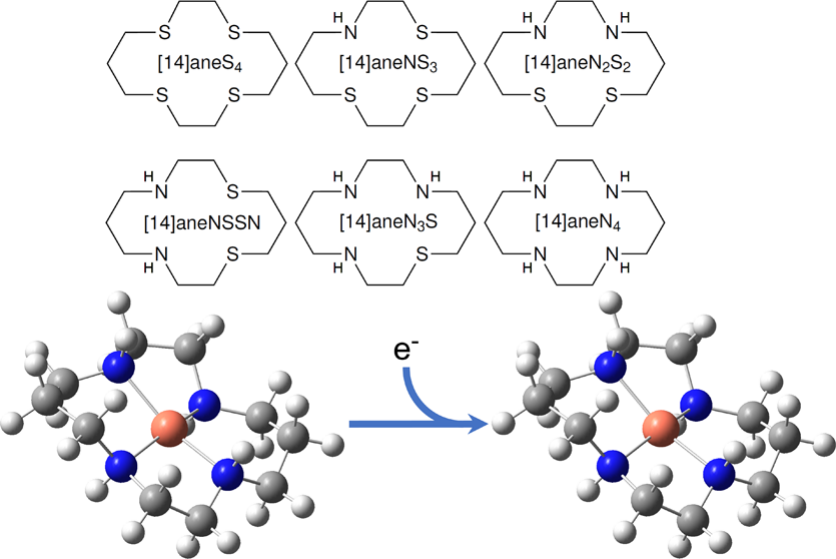

Suitability of single-reference density functional theory (DFT) methods for the calculation of redox potentials of copper-containing macrocycle complexes was confirmed by the use of *T*_1_ diagnostics along with a verification of negligible spin contamination or wave function instability. When examining the effect of improvement in the cc-pV*n*Z basis set series on calculated redox potentials, the results readily converged at the cc-pVTZ level. The all-electron Def2-TZVPP basis set is shown to be a suitable choice of a basis set for the calculation of redox potentials when utilizing a cc-pVTZ geometry. The best-performing model chemistries are determined to be the M06/polarizable continuum model (PCM); therefore, a scheme for redox potential calculations of copper macrocycles using either M06/cc-pVTZ with PCM solvation is proposed to reliably reproduce experimental trends.

## 1 Introduction

Many fundamental chemical reactions include steps involving electron transfer from one species to another. The prominence of these electron-transfer events is commonly described, utilizing reduction and oxidation (redox) potentials and thermodynamic properties that quantitatively describe the tendency of chemical compounds to lose or acquire electrons. Experimentally, these properties are determined through the use of techniques such as cyclic voltammetry, which allows the measurement of redox potentials for reversible electron-transfer processes. However, the experimental determination of redox potentials for nonreversible reactions is more complicated, requiring the use of rapid spectrophotometric techniques such as pulse radiolysis to obtain reliable values.^1^ Computational approaches can be used to enhance the descriptions provided by the experimental data, capturing information hidden due to the complex nature of the experimental setup. This can be achieved through the ability to separate various effects and break down the structural complexity of a reaction, allowing focus on, for example, structure–energy relationships.

A subset of these redox reactions that are of significant biochemical importance are those involving copper complexes, which play an essential role in a number of living systems and are commonly separated into four types, depending on the number of metal center, oxidation state, and coordination environment.^2,3^ Each of these complexes is involved in various key roles, including electron transfer, O_2_ binding, and various enzymatic reactions.^4,5^ Therefore, a detailed understanding of the redox chemistry of copper-binding complexes can provide deeper insight into the mechanisms of copper metalloproteins and copper trafficking, which is important in the regulation of normal human physiology and homeostasis,^6,7^ with the highest concentration of such proteins being found in the liver and brain.^8^ Moreover, redox properties of copper-binding drugs, such as metformin,^9^ may play a crucial role in the biomolecular function of these drugs as they can, for example, interfere with the sensitive redox chemistries occurring inside the cell, such as the mitochondrial electron transport chain. Additionally, the dedicated use of these copper-chelating compounds has been investigated as a potential treatment for a range of physiological and neurological conditions due to the cytotoxic effects of increased copper concentrations caused by dietary intake, environmental factors, or a failure in biological mechanisms for the removal of excess copper.^10^ The effects of this excess, regardless of cause, can result in an increased production of reactive oxygen species (ROS), leading to heavy oxidative stress on the cellular environment.^11−13^

The effect of coordination geometry, the nature of the donor atoms of the ligands, and a number of substituents have been shown to affect the redox potential of the Cu^II^/Cu^I^ couple.^14−16^ For example, there is a dependence of the Cu^II/I^ redox potential on the relative number of N and S donor atoms, mainly guided by the Cu^II^ preference for amine nitrogen relative to thioether sulfur, while Cu^I^ shows a preference for sulfur coordination.^2,17^ Moreover, the reduction of Cu^II^ to Cu^I^ is facilitated in the case of ligands containing both unsaturated nitrogen and thioether sulfur atoms.^18^

Within a biological environment, Cu^I/II^ ions are coordinated predominantly, and with a few exceptions, by histidine, cysteine, and methionine residues through their N and S atoms. Despite the limited number of coordination residues, their combination provides a high degree of flexibility and diversity in the structure of the Cu^I/II^ coordination sphere.^19^ Given these coordination options, the ability to accurately determine and quantify the effect of N/S coordinating atoms on the redox properties of a given Cu^I/II^ center can be pivotal in developing an understanding of the enzymatic activity and how increased cytoplasmic copper concentrations can be mitigated to maintain homeostasis and as a treatment for oxidative stress.^10−13^ Density functional theory (DFT), in conjunction with continuum solvation models, has been successfully applied in studies involving the prediction of redox potentials of organic molecules, such as anilines^20^ and polycyclic aromatic hydrocarbons^21^ and transition-metal complexes such as ferricinium/ferrocene couples^22,23^ and copper complexes.^24,25^ Marenich et al.^26^ review further examples of the application of DFT and other computational methods in calculations of reduction potentials.

In spite of encouraging results from DFT calculations, investigations of redox potentials often lack a more systematic approach to understanding the fundamental elements of the redox potential calculation in depth. Moreover, the use of a huge variety of different functional and basis set combinations across the literature and insufficient benchmarking may lead to an unnecessarily difficult task for a new user to calculate redox potentials and assess their accuracy.

In general, redox potential calculations of transition-metal complexes present a challenge due to the usually large, structural differences between the oxidized and reduced states of the complex, excess charge of the complex, and potential multireference character of the transition-metal wave function. Some of these issues can be addressed by the use of higher-level methods or the use of multiple conformers. Matsui et al.^27^ proposed a scheme to address the metal complexes with excess charge by putting an image counterion distribution around the charged complex to neutralize the system and improve the often poor description of the solvation energy.

One of the first studies involving the prediction of redox potentials of organic molecules was the work done by Winget et al.,^20^ who used the B3LYP hybrid functional and the cc-pVTZ(-f) basis set. They studied substituted anilines and determined that linear relationships between theoretical predictions and experiment are constructed and provide mean unsigned errors as low as 0.02 V over a training set of 13 anilines; the error increases to 0.09 V over a test set of eight additional anilines. A good correlation is also found between oxidation potential and a simple computed property, namely, the energy of the highest occupied molecular orbital for neutral anilines in aqueous solution.

**Figure 1:**
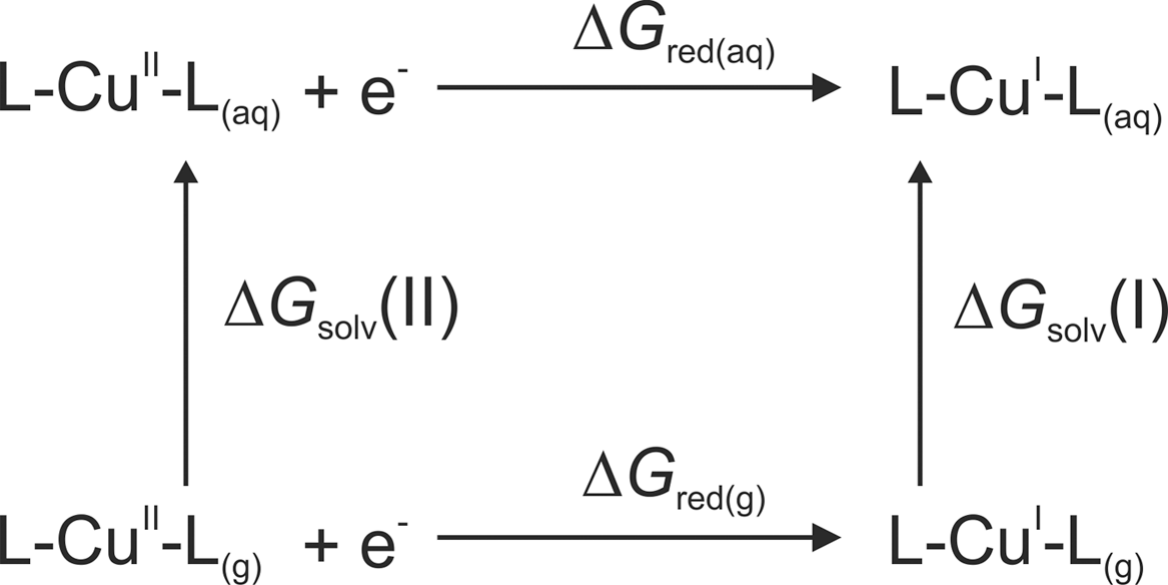
Born–Haber cycle for the calculation of redox potential.

Due to the initial electron-transfer step often acting in the rate-determining step for a given reaction, and the correlation between the rate constant and energetics, a key molecular descriptor in modeling electron-transfer kinetics is the one-electron redox potential.^20^ Although levels of theory that do not demand large amounts of computational resources, such as DFT, can be inaccurate for ionization potentials and/or free energies of aqueous species, thereby leading to errors in directly calculated absolute oxidation potentials, these errors are sometimes systematic, in which case a linear correction scheme can be efficacious. Recalling that theory is being used to compute the top, left, and right sides of the free-energy cycle in Figure 1, and noting that the predicted errors are commonly equivalent, regardless of whether theoretical or experimental IPs are used, we must conclude that the problem lies in the computation of the solvation free energies. Furthermore, since the solvation models provide an accurate prediction for the known solvation free energy of aniline, and since the total variation in solvation free energy over the neutrals is small, it appears likely that the error is in the computation of the solvation free energy for the aniline radical cations.

**Figure 2:**
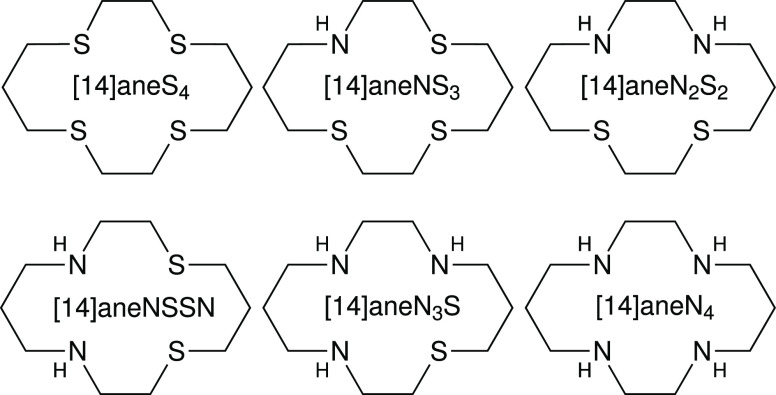
Macrocyclic ligands used in the reduction potential study.

Here, a systematic computational protocol for the calculation of reduction potentials of copper complexes is developed and tested on the series of 14-membered quadradentate macrocyclic polyamino polyether ligands (Figure 2). These molecules represent a series of valid, and experimentally characterized, copper-binding model systems, previously used as a model for blue copper-binding sites.^28,29^ Moreover, the use of this series enables a thorough examination of the effect of thioether sulfur substitution for amine nitrogen on the electrochemical properties. Although, in the original experimental paper by Rorabacher,^17^ these macrocyclic complexes serve as model compounds for blue copper protein binding sites, their application as model systems can be potentially extended to other important copper-binding complexes (*e.g.*, metformin copper complex). The specific use of these six model compounds allows for the isolation of coordination sphere effects due to the lack of additional electronic groups throughout each macrocycle and a ring size large enough to allow for flexibility in the coordination geometry with minimal strain induced by the CH_2_ link groups. This allows for the accurate investigation of diverse, biorelevant, coordination environments in a systematic manner.

Experimental reduction potentials, listed in Table 1, are obtained from Rorabacher et al.^17^ and were determined using cyclic voltammetric measurements on aqueous Cu^II^L solutions at 25 °C. For the empirical estimation of potential values of the N_3_S complex, the following equation was used^17^
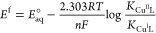
1where the *E*_aq_^°^ is the standard electrode potential, in terms of molar concentrations, for the Cu_aq_^2+^/Cu_aq_^+^ couple of the aqueous Cu^II/I^. In the original ref (17), it was measured as *E*_aq_^°^ ≈ 0.13 V at 25 °C.

**Table 1:** Experimental *E*_1/2_ Potentials for Studied N_*x*_S_4–*x*_ (*x* = 0, 1, 2, 3, 4) Complexes

N_*x*_S_4–*x*_	*E*_1/2_^a^ [mV]
N_4_	–0.66 (est)^b^
N_3_S	⩽−0.24 (est)^c^
N_2_S_2_	0.04 (pH > 5.0)
NSSN	–0.01 (pH > 5.0)
NS_3_	0.38 (pH > 3.5)
S_4_	0.58

a Experimentally determined *E*_1/2_ should be accurate to within ±0.01 V.^17^^17^

b Values estimated from the trend of methanolic potentials of copper complexes with related 14-membered macrocyclic N_4_ ligands containing unsaturated nitrogen^33^ assuming *E*_H_2_O_^f^ = *E*_H_2_O_^MeOH^ – 0.060 V.^34,35^

c Value estimated using eq 1 assuming *K*_Cu^II^L_ ≥ 1 × 10^20^ and *K*_Cu^I^L_ ≈ 4 × 10^13^.

## 2 Computational Electrochemistry

Determination of equilibrium redox potentials makes use of a Born–Haber cycle (Figure 1), which represents the free-energy changes observed in the solvent phase (top) and the gas phase (bottom); the cycle is completed by taking into account the solvation free energies of each species (left and right). The use of this cycle circumvents the need to calculate the energy of the solvated free electron through the neglect of the free electron energy in the gas-phase free-energy change (at 0 K, this would typically correspond to an electron affinity (EA) as an analog for reduction potential and an ionization potential (IP) for oxidation potential) and free energies of solvation. This enables the solvated reduction potential (Δ*G*_red(aq)_) to be calculated such that
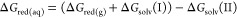
2where the gas- and solvent-phase terms (Δ*G*_red(g)_ and Δ*G*_solv_, respectively) were calculated as a difference between electronic energies at the relevant geometric minima in the implicit solvent and gas-phase environments, such that
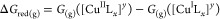
3
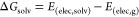
4Further, a correction to the gas-phase Gibbs energy of reaction, ≈1.89 kcal/mol, placing it from the initial reference state of 1 atm to 1 mol/L, is included in the calculation. However, neglecting this correction would lead to a relatively small error of 80 mV to the calculated absolute potentials.

Once determined, Δ*G*_red(aq)_ is then used to calculate the absolute half-cell standard reduction potentials *E*_Abs_^0^ using the equation
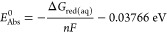
5where −0.03766 eV represents a free electron correction at 298 K,^30^*F* is the Faraday constant (23.06 kcal/mol·V), and *n* represents the number of electrons involved in the redox couple.

Absolute reduction potentials are reported as well as relative potentials referenced to a standard hydrogen electrode (SHE). To reference against SHE, a suggested absolute SHE potential value of +4.28 V,^31^ which is in good agreement with an experimentally determined value of +4.29 V,^32^ is subtracted from the calculated absolute potentials.

## 3 Methods

Equilibrium geometries of all of the computed structures were obtained by gas-phase optimization at the DFT level of theory using various functionals, and each structure was verified to be a true minimum by the absence of imaginary frequencies in the vibrational analysis. The appropriateness of each geometry was assessed against a range of experimental structures^17,36−40^ (Tables S1–S36). Energy differences for Δ*G*_red(g)_, Δ*G*_solv_(I), and Δ*G*_solv_(II) eq 2 were determined through the comparison of energy of each structure at the geometric minima, as determined through optimization in either the gas phase or the relevant solvent model, respectively.

For each calculation, the stability of the wave function was tested. Basis sets used were Def2-TZVPP^41^ and cc-pVTZ^42,43^ (Table 2). All calculations were conducted using all-electron variants of each basis set. Calculations were performed using Gaussian09 (A.02 version).^44^ Implicit solvent calculations were computed using the standard self-consistent reaction field (SCRF) approach with polarizable continuum model (PCM),^45−49^ the integral equation formalism variant (IEFPCM), and SMD^50^ solvation model, and water as a solvent.

**Table 2:** Absolute Deviations of Calculated Reduction Potentials (mV) from the Experimental Values (Table 1), *i.e.* |(*E*_calc_ – *E*_exp_)|, for the M06 Functional, Utilizing the cc-pVTZ and Def2-TZVPP Basis Sets with SMD Solvation Model

N_*x*_S_4–*x*_	cc-pVTZ	Def2-TZVPP
N_4_	0.74	26.60
N_3_S_1_	45.24	30.39
N_2_S_2_	89.14	67.76
NSSN	20.00	7.32
N_1_S_3_	69.34	68.47
S_4_	88.32	49.90
MAD	52.13	49.24

Calculations were carried out using the following functionals SVWN,^51−54^ BP86,^55,56^ B3LYP,^54,57−59^ B3PW91,^54,57,60−63^ CAM-B3LYP,^64^ B97D,^65^ ωB97xD,^66^ TPSSTPSS,^67^ PBE1PBE (PBE0),^68−70^ and Minnesota functionals with different % HF exchange included (in parentheses): M06-L^71^ (0%), M06^72^ (27%), M06-2X^72^ (54%), and M06-HF^73^ (100%).

SVWN is a local spin density functional with the Slater exchange, ρ^4/3^ with a theoretical coefficient of 2/3, and Vosko, Wilk, and Nusair 1980 correlation functional (III) fitting the RPA solution to the uniform electron gas. BP86 represents a generalized gradient approximation (GGA) functional, which incorporates Beckes 1988 exchange functional B with Perdew 1986 correlation functional P86, family of functionals. B3LYP is the hybrid functional, which incorporates Beckes three-parameter exchange functional B3 with the Lee, Yang, and Parr correlation functional LYP. The B3PW91 is similar to the B3LYP functional, but the nonlocal correlation is provided by Perdew/Wang 91. B97D is the GGA exchange–correlation functional including dispersion. TPSSTPSS represents the τ-dependent gradient-corrected functional, and PBE1PBE (PBE0) is the hybrid functional that uses 25% exact exchange and 75% DFT exchange. Minnesota functionals, containing the fully local meta-GGA M06-L functional, account implicitly for dispersion effects and should perform well for systems containing transition metals. In the same family of functionals, M06, M06-2X, and M06-HF represent the global hybrid functionals. In addition, the inclusion of Grimme’s dispersion, both with and without the Becke–Johnson damping,^74^ was tested with the B3LYP and B97 functionals. In the above functionals, the long-range electron–electron exchange part typically dies off too rapidly and becomes very inaccurate at large distances, making these functionals unsuitable for modeling processes such as electron excitations and charge-transfer states. Long-range corrected functionals such as ωB97xD and CAM-B3LYP were designed to address these problems by separating the two-electron operator, , into the short-range and long-range parts using the standard error function erf. The commonly used CAM-B3LYP functional uses 19% exact HF and 81% Beckes 1988 exchange interaction at short range and 65% HF and 35% Beckes 1988 at long range. The intermediate region is smoothly described by the parameter μ = 0.33, controlling the partitioning of the interelectronic distance.

### 3.1 Assessing Appropriateness of DFT for Redox Potential Calculations

To assess the reliability of the single-reference DFT for the reduction potential calculations of the copper macrocycles, the multireference character of the wave function was assessed using *T*_1_(75−78) diagnostic values calculated at the CCSD/cc-pVDZ level, and spin contamination was assessed through the expectation value of the total spin ⟨S^2^⟩ and the wave function stability.^79−81^

The *T*_1_ diagnostics, which uses the Frobenius norm of the *t*_1_ amplitudes of the CCSD wave function, provide an averaged indicator of the quality of a single-reference coupled cluster but may fail to indicate a small problem region of a large molecule. A criterion of *T*_1_ > 0.05 was proposed to identify 3d transition-metal species with substantial nondynamical correlation, for which results obtained from a single-reference quantum method may suffer from large errors and unpredictable behavior.^82^ The *T*_1_ diagnostic has not been used on many large copper complexes, and so data is limited to only small copper complexes. In general, the *T*_1_ values for small copper complexes tend to be lower than 0.05, with only a small number of cases denoting large *T*_1_ values noted in the literature and involving small, coordinately unsaturated species where a multireference character would be expected.^82,83^ For the macrocyclic complexes presented in this chapter, the *T*_1_ diagnostic was calculated for completeness as it is readily available from the final coupled cluster (CC) wave function; however, there is no a priori reason to expect large values.

The spin contamination is a result of unrestricted wave function no longer being a pure eigenfunction of the total spin ⟨S^2^⟩ and therefore the desired spin state may suffer interference from other spin states, which may result in some errors, *e.g.*, increase in the total energy.

Finally, the stability test ensures that the resulting single-determinant wave function is a local minimum with respect to relaxing various constraints, *e.g.*, allowing a restricted Hartree–Fock (RHF) determinant to become unrestricted Hartree–Fock (UHF), allowing orbitals to become complex and reducing the symmetry of the orbitals.

## 4 Results and Discussion

### 4.1 Appropriateness of DFT

Examining the *T*_1_ values in Table 3, all of the studied complexes and copper oxidation states exhibit *T*_1_ values lower than the 0.05 criteria and thus can be assumed to provide a reasonably accurate description of the wave function. There is a notable increase in *T*_1_ values within the N_*x*_S_4–*x*_ series for Cu^II^ as the number of sulfurs present in the ring increases. Additionally, the inclusion of an implicit solvent model consistently decreases the *T*_1_ value, with minimal difference observed between solvent models.

**Table 3:** Multireference Character Measured Using *T*_1_ Diagnostics Values^a^

	Cu^II^			Cu^II^		
N_*x*_S_4–*x*_	Gas	PCM	SMD	Gas	PCM	SMD
N_4_	0.0206	0.0194	0.0183	0.0158	0.0158	0.0161
N_3_S_1_	0.0228	0.0213	0.0213	0.0154	0.0153	0.0154
N_2_S_2_	0.0246	0.0230	0.0231	0.0152	0.0150	0.0155
NSSN	0.0252	0.0235	0.0234	0.0154	0.0153	0.0152
N_1_S_3_	0.0281	0.0264	0.0262	0.0153	0.0152	0.0152
S_4_	0.0311	0.0294	0.0295	0.0153	0.0152	0.0152

a Calculated at the CCSD/cc-pVDZ level.

*T*_1_ values for Cu^I^ complexes are consistently lower than their Cu^II^ counterparts, which, due to the singlet nature of these complexes, is unsurprising; there is also negligible effect observed upon inclusion of a solvent model.

Looking at the eigenfunctions of the total spin ⟨S^2^⟩ for all of the unrestricted calculations, it can be concluded that a spin contamination, if present, was completely removed after a spin annihilation step and the calculated wave functions represent pure spin states, ⟨S^2^⟩ = 0.75 (Cu^II^). Lastly, the stability tests showed that none of the calculations suffered from any stability problems.

### 4.2 Dependence on Functional and Solvent Models

**Figure 3:**
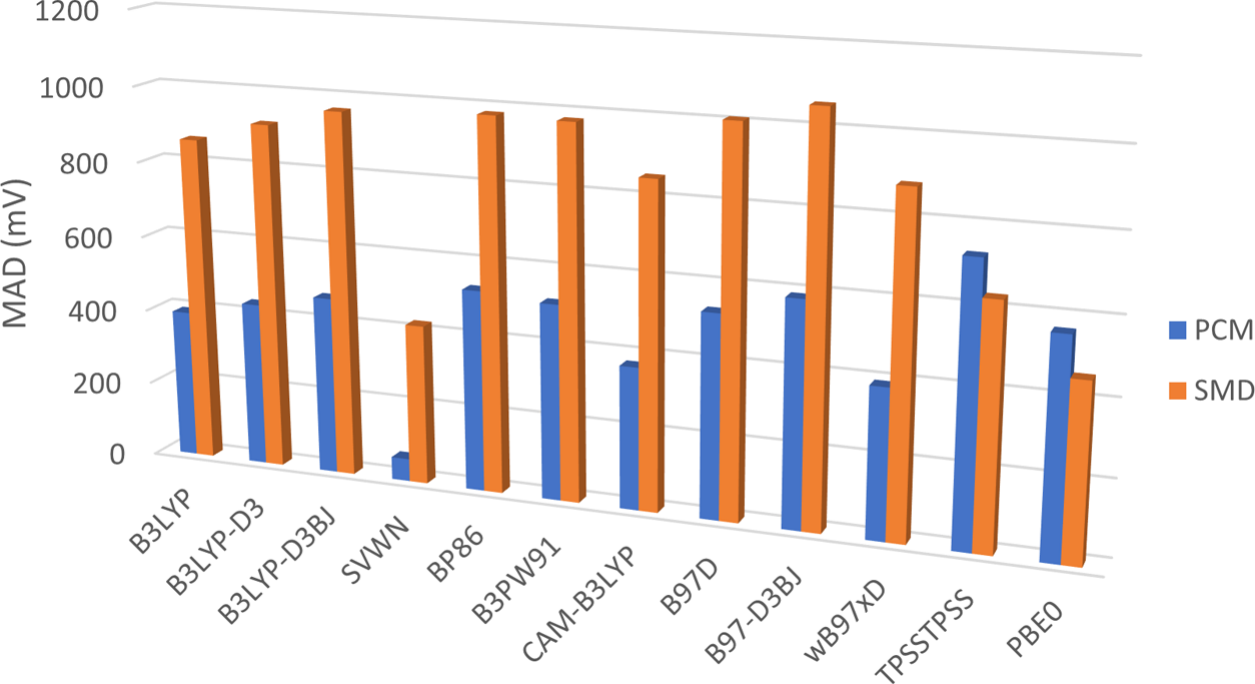
Mean absolute deviation (mV) of theoretical values from experimental values for different functionals using the cc-pVTZ basis set.

**Figure 4:**
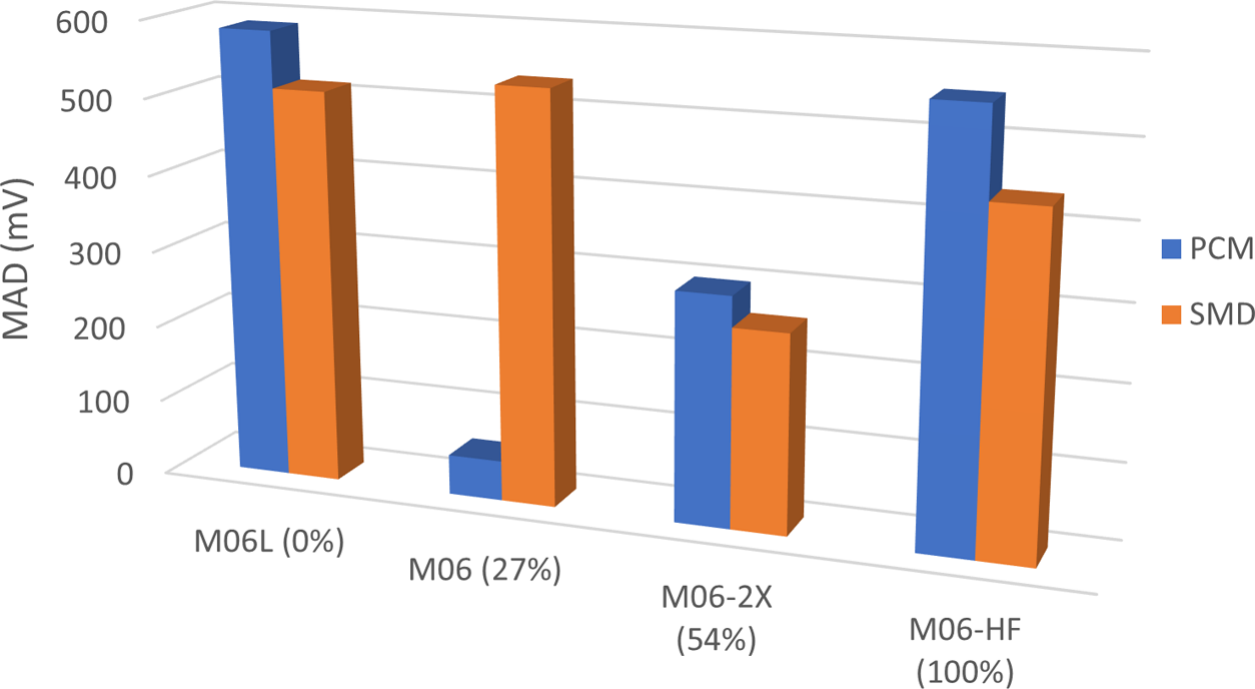
Mean absolute deviation (mV) of theoretical values from experimental values for the M06 family of functionals, utilizing the cc-pVTZ basis set, in ascending order of %HF exchange (in parentheses).

A high degree of variance is observed in the performance of the functionals studied (Figures 3 and 4), with the combination of the M06 functional and the PCM solvent model with an MAD = 52 mV (Figure 4), closely followed by SVWN coupled, again, with the PCM solvent model (MAD = 60 mV; Figure 3).

Across the majority of functionals, the PCM solvent model produces a smaller MAD value compared to equivalent calculations utilizing the SMD model; in those cases in which SMD outperforms the PCM model (*e.g.*, TPSSTPSS and PBE0; Figure 3), the difference in performance was significantly reduced compared to functionals for which PCM performed better (*e.g.*, B3LYP and SVWN; Figure 3). Additionally, with the exception of M06, all other members of the M06 family (Figure 4) perform better with the SMD model.

Inclusion of any correction in the form of empirical dispersion is shown to significantly reduce the accuracy of each functional (Figure 3), irrespective of the solvent model applied. Application of the Becke–Johnson damping is also shown to further reduce the accuracy of a given calculation when compared to both the correction-free and dispersion-only variations.

### 4.3 Effect of N for S Substitution

**Figure 5:**
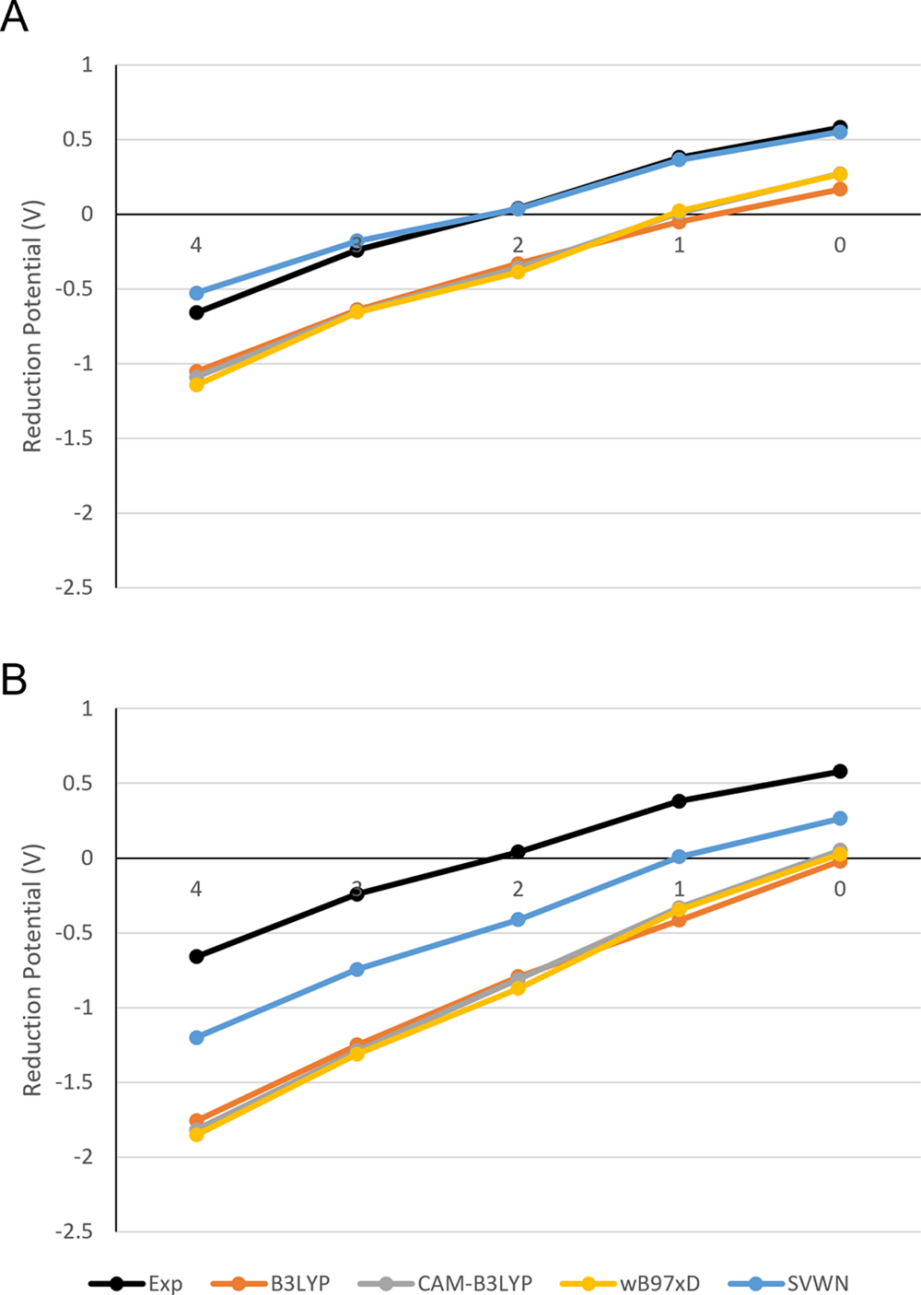
Comparison of experimental reduction potentials with a selection of the best-performing functionals tested for the N_*x*_S_4–*x*_ series in the PCM (A) and SMD (B) solvent models. Note that NSSN was omitted for simplicity from this comparison as its redox potential is close to that of N_2_S_2_.

**Figure 6:**
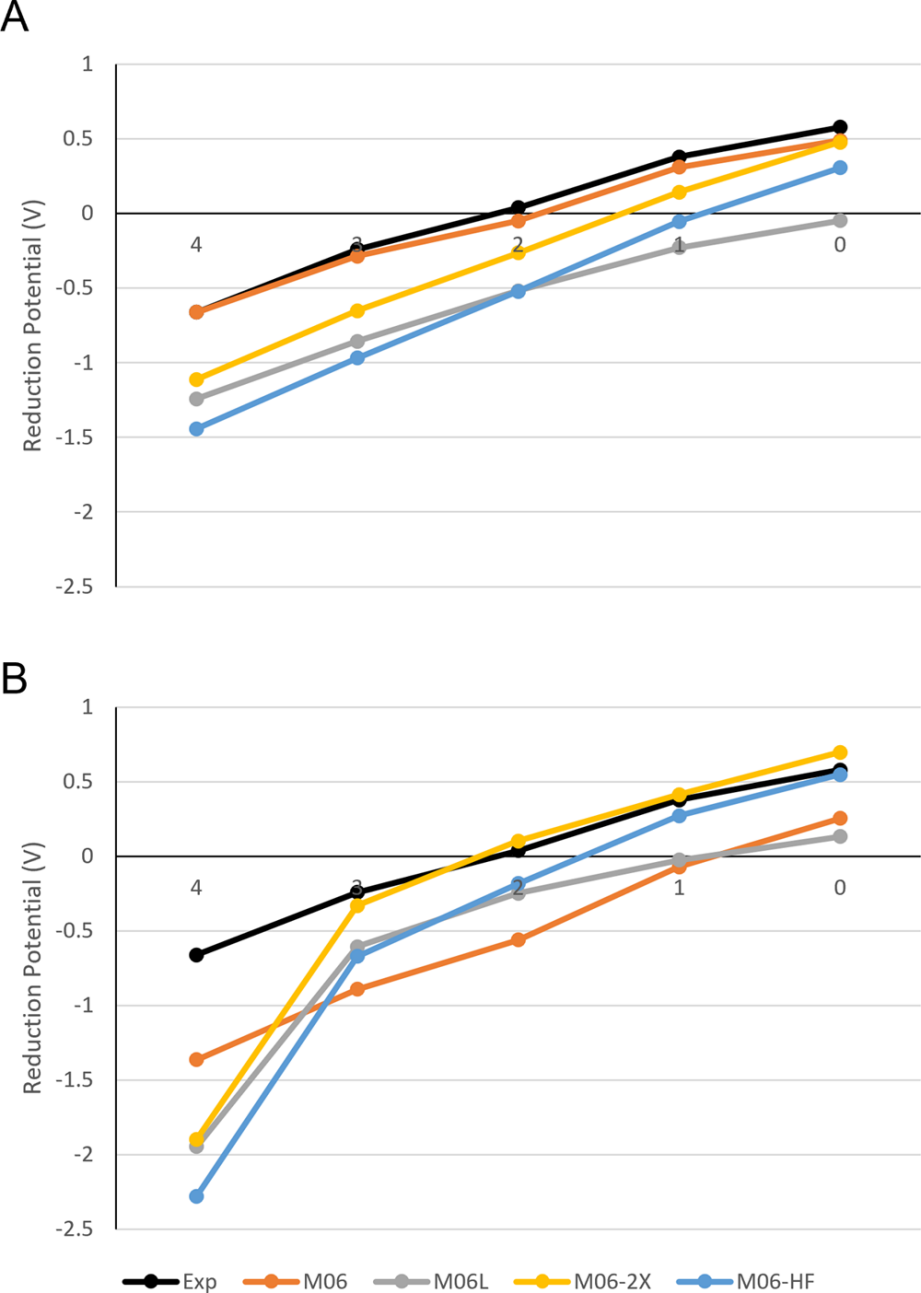
Comparison of experimental reduction potentials with the M06 family of functionals tested for the N_*x*_S_4–*x*_ series in the PCM (A) and SMD (B) solvent models. Note that NSSN was omitted for simplicity from this comparison as its redox potential is close to that of N_2_S_2_.

The four best-performing functionals (Figure 5) and the M06 family of functionals (Figure 6) were investigated to assess the accuracy of each model chemistry in determining the reduction potential for each of the structures studied.

With the exception of the combination of the SMD solvent model with the M06 family (Figure 5B), each of the other model chemistries is seen to perform equivalently on each structure within the series. This consistent behavior lends further confidence to the use of MAD values (Figures 3 and 4) in assessing the performance of a given functional.

The application of SMD to the M06 family presents a more varied performance with M06-L, M06-2X, and M06-HF all producing significantly worse results for N_4_ than for all other structures. Removal of this structure from the MAD calculations results in a reduction of the MAD value of M06-2X from 258 to 52 mV, equivalent to that observed with M06//PCM.

### 4.4 Basis Set Dependence of Calculated Redox Potentials

**Figure 7:**
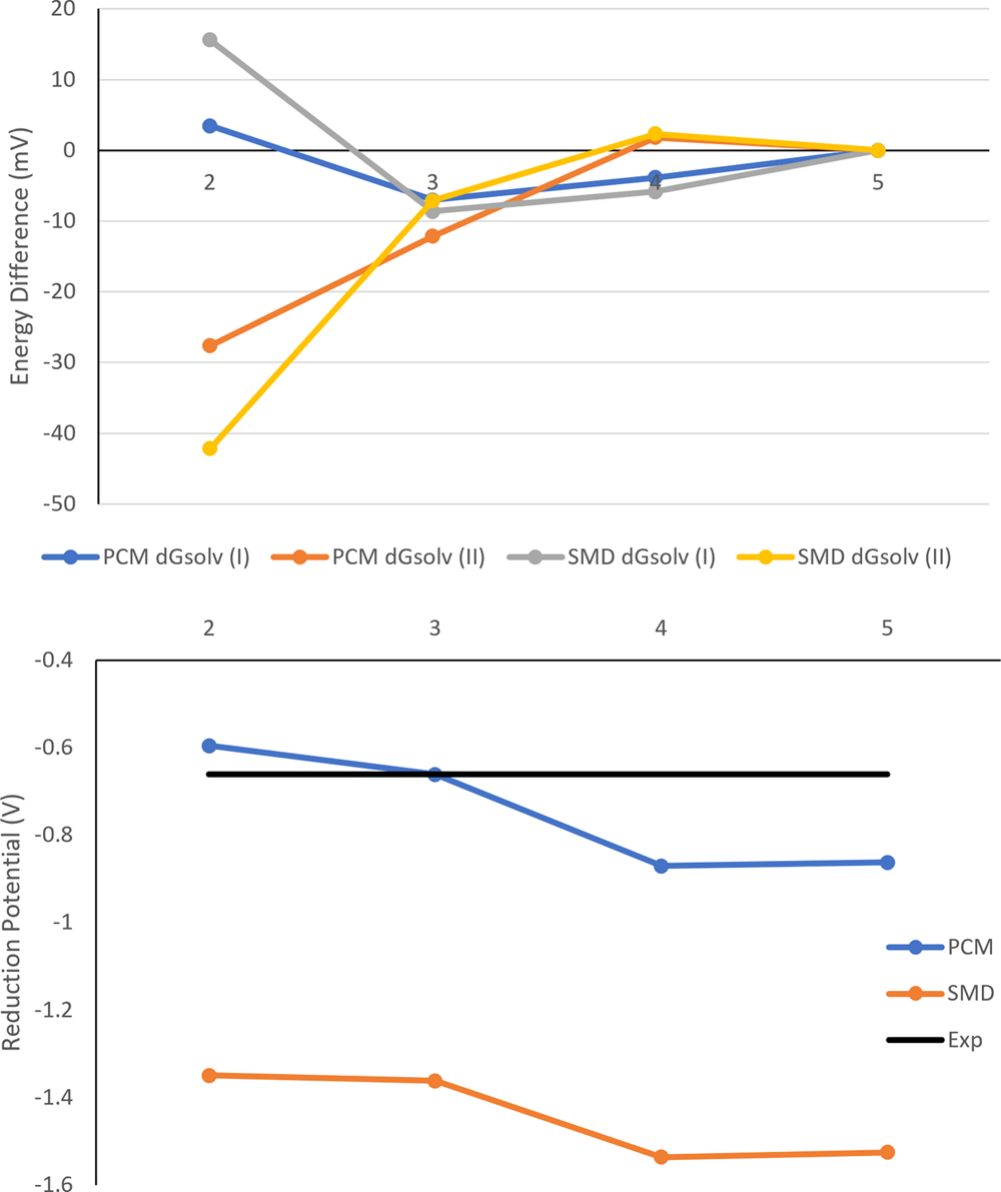
Convergence of Δ*G*_solv_(I) and Δ*G*_solv_(II) of lower basis sets to the cc-pV5Z values (top) and calculated reduction potentials (bottom) with the cc-pV*n*Z basis set for the N_4_ complex.

Analysis of the calculated reduction potentials at increasing basis set sizes for the N4 structure (Figure 7; bottom) further acts to verify the more favorable performance of the PCM solvent model over the SMD model, with a similar difference (≈800 mV) observed regardless of the basis set. This trend is observed across all structures studied (see the Supporting Information) and is further highlighted by considering the MAD values for each combination of basis set and model chemistry (Table 4) where a constant difference of ≈450 mV is observed.

**Table 4:** Effect of Basis Set Improvement on the MAD (mV) of Calculated Reduction Potentials

cc-pV*n*Z	PCM	SMD
2	35	491
3	52	536
4	180	647
5	176	642

Figure 7 and equivalent analysis for the rest of the N_*x*_S_4–*x*_ series (see the Supporting Information) also suggest that the cc-pVQZ and cc-pV5Z basis sets perform significantly worse than the cc-pVDZ and cc-pVTZ basis sets; this observation is most likely a result of the reduced fortuitous cancellation of errors as the basis sets improve. An additional source of this surprising deviation may also be attributed to the use of the cc-pVTZ free-energy corrections in combinations of higher-level basis sets. However, the necessary frequency calculations to determine the basis set-specific free-energy correction for higher-level basis sets would render the calculations intractable for even these relatively small complexes.

Investigation of the individual components of the Born–Haber cycle (Figure 7; top): a notable difference is observed in the cc-pVDZ deviations and in the cc-pVTZ basis set; meanwhile, the deviation of the cc-pVTZ and cc-pVQZ from the cc-pV5Z values is significantly smaller. These observations further suggest that the performance of each basis set may, to a significant degree, be driven by the determination of the free-energy corrections, given that the TZ/QZ/5Z models all share the same correction value.

### 4.5 Geometry Dependence

Comparison of experimental geometries taken from crystal structures^17,36−40^ with the average values determined by the DFT functionals (see the Supporting Information) shows reasonable agreement in the metal–ligand bond length; however, the characterization of L–M–L bond angles shows less agreement. The lesser agreement observed in the calculated angles can be accounted for due to each of the referenced crystal structures possessing a five-coordinate Cu^II^ center, resulting in a larger angle than the four-coordinate Cu^II^ and Cu^I^ centers utilized during calculations. The largest of these deviations is found in the N4–Cu^II^ structure, with a difference of ≈19° between the average performance of DFT functionals and the experimental crystal structure.

**Figure 8:**
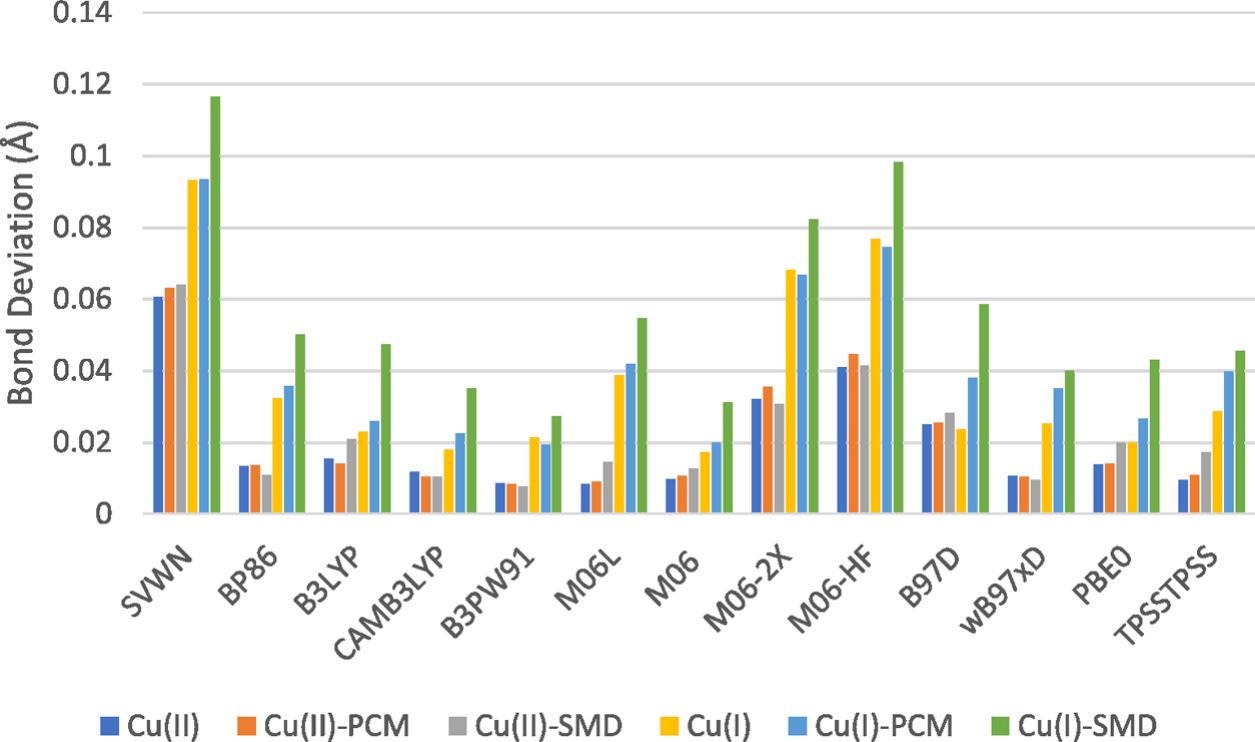
Deviation of M–L bond lengths from the average across all functionals.

**Figure 9:**
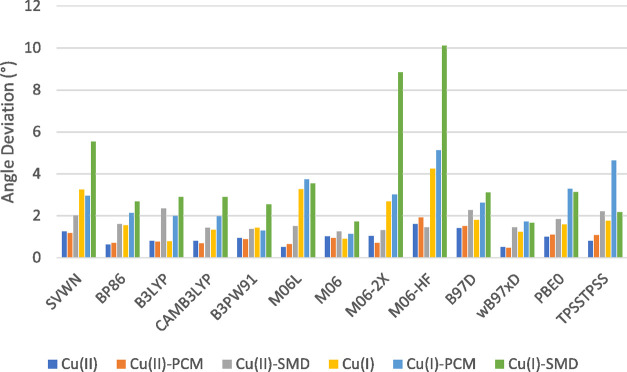
Deviation of L–M–L angles from the average across all functionals.

Deviations of calculated values of each functional from the average DFT value are shown for bond lengths (Figure 8) and angles (Figure 9). When considering both bond length and angle values, the deviation from the mean is significantly lower for Cu^II^ structures than their ^I^ counterparts. Deviations in bond length for Cu^II^ structures are observed to be less than 0.02 Å for most functionals; exceptions to this are seen in the SVWN, M06-2X, and M06-HF functionals, with SVWN showing the largest deviation at ≈0.06 Å. Consideration of Cu^I^ shows a similar trend, with SVWN showing the largest deviation at ≈0.12 Å, followed by M06-2X and M06-HF; however, it is worth noting that the difference in agreement between PCM and SMD values is significantly larger for Cu^I^ structures than observed with Cu^II^.

A similar trend can be observed in the agreement of angles (Figure 9), with Cu^II^ structures shown to be under 2° across most structures. Model chemistries of note are observed again, with the SVWN, M06-2X, and M06-HF functionals showing deviations of 6, 9, and 10°, respectively.

Despite the deviations observed in calculations utilizing the SVWN, M06-2X, and M06-HF functionals, the overall agreement is still high when compared to both the DFT average and the experimental values. This suggests that the source of variation observed in redox potential cannot be attributed to the geometry produced by each functional; instead, it is most likely a product of the differences in energetic components and Gibb’s free-energy corrections of each Born–Haber cycle.

## 5 Conclusions

The effects of coordination environment on the redox activity of biorelevant copper complexes were systematically investigated throughout this work, alongside the performance of a broad range of single-reference density functionals.

The suitability of these single-reference DFT methods for the calculation of redox potentials of copper-containing macrocycle complexes was confirmed by the use of *T*_1_ diagnostics along with a verification of negligible spin contamination or wave function instability. Additionally, the effect of functional choice on geometry and redox potential was verified through deviation from crystal structures and experimental potentials, respectively.

When examining the effect of improvement in the cc-pV*n*Z basis set series on calculated redox potentials, the results readily converged at the cc-pVTZ level. The all-electron Def2-TZVPP basis set is a suitable choice of a basis set for the redox potential calculations where the inclusion of some diffuse functionality is desired in the basis set while also potentially leading to smaller absolute deviations from the experimental redox potential.

While the geometrical agreement between functionals is high, the reduced performance of SVWN compared to that of the other functionals studied and the large deviations observed in the Cu^II^-SMD structures, this further suggests the use of M06//PCM model chemistry to evaluate redox potentials for similar systems.

Summarizing the above findings in the proposal of a suitable scheme for redox potential calculations of copper macrocycles involves the use of the M06//cc-pVTZ model chemistry while utilizing the PCM solvent model for relevant structures.

Future work would involve the in-depth investigation of functional and basis set choices on various properties, for example, implicit solvent cavity shape/size and highest-occupied molecular orbital/lowest-unoccupied molecular orbital (HOMO/LUMO) gap.^21,84^ Moreover, the use of other methods to obtain more accurate electron affinities (ionization potentials) and free energies of solvation should be considered such as the use of an electron propagator theory methodology for the calculation of correlated electron affinities and ionization potentials.^85,86^

## Supporting Information Available

Geometric parameters (M–L bond lengths and L–M–L angles) calculated for each complex in addition to basis set convergence analysis for complexes N_*x*_S_4–*x*_ (*x* = 1–3) (PDF)
