# Supplementary material for: Protocols for Understanding the Redox Behavior of Copper-Containing Systems
Source: ACS Omega. 2022 Nov 30;7(49):45057–66. doi: 10.1021/acsomega.2c05484 (PMC9753522; doi:10.1021/acsomega.2c05484)
Supplement: Supplementary file 1 — ao2c05484_si_001.pdf [file ao2c05484_si_001.pdf]

Protocols for Understanding the Redox Behaviour of Copper Containing Systems - Supplementary Information

Thomas Malcomson,*,† Peter Repiščák,‡ Stefan Erhardt,¶ and Martin J. Paterson*,§

†*Department of Chemistry, School of Natural Sciences, The University of Manchester, Manchester, M13 9PL, UK.*

‡*Beatson Institute for Cancer Research, University of Glasgow, Garscube Estate Switchback Road, Bearsden, G61 1QH, UK.*

¶*School of Life, Sport and Social Sciences, Edinburgh Napier University, Edinburgh, Scotland, UK.*

§*Institute of Chemical Sciences, School of Engineering and Physical Sciences, Heriot-Watt University, Edinburgh, EH14 4AS, UK.*

E-mail: thomas.malcomson@manchester.ac.uk; m.j.paterson@hw.ac.uk

References

(1) Antsyshkina, A. S.; Porai-Koshits, M. A.; Makhaev, V. D.; Borisov, A. P.; Kedrova, N. S.; Mal'tseva, N. N. Synthesis and crystal structure of (1,10-phenanthroline)(triethyl phosphite)copper(I) tetrahydroborate. *Koord.Khim.(Russ.)(Coord.Chem.)* **1992**, *18*, 474–480.

(2) Lindoy, L. F.; Mahinay, M. S.; Skelton, B. W.; White, A. H. Ligand assembly and metal ion complexation: syntheses and X-ray structures of Ni(II) and Cu(II) benzoate and 4-tert-butylbenzoate complexes of cyclam. *J. Coord. Chem.* **2003**, *56*, 1203–1213.

(3) Hörmann, E.; Riesen, P. C.; Neuburger, M.; Zehnder, M.; Kaden, T. A. Metal Complexes with Macrocyclic Ligands. Part XLI. Nickel(II) and copper(II) complexes with mono-N-functionalized dithiadiazamacrocycles. *Helv. Chim. Acta.* **1996**, *79*, 235–243.

(4) Bernardo, M. M.; Heeg, M. J.; Schroeder, R. R.; Ochrymowycz, L. A.; Rorabacher, D. B. Comparison of the influence of saturated nitrogen and sulfur donor atoms on the properties of copper (II/I)-macrocyclic polyamino polythiaether ligand complexes: Redox potentials and protonation and stability constants of CuIL species and new structural data. *Inorg. Chem.* **1992**, *31*, 191–198.

(5) Desper, J. M.; Gellman, S. H. Crystallographic evidence for multiple coordinating conformations in a family of macrocyclic chelators. *J. Am. Chem. Soc.* **1991**, *113*, 704–706.

(6) Glick, M. D.; Gavel, D. P.; Diaddario, L. L.; Rorabacher, D. B. Structure of the 14-membered macrocyclic tetrathia ether complex of copper(II). Evidence for undistorted geometries in blue copper protein models. *Inorg. Chem.* **1976**, *15*, 1190–1193.

1 Geometries and Basis Set Convergence

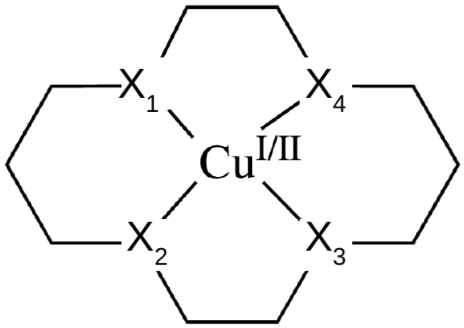

Figure S1: Illustrative drawing of labelling system for the critical bonds and angles of the X-ray and computed structures. The bond angles are defined between the X_1-Cu-X_3 and X_2-Cu-X_4.

Table S1: Comparison of critical bonds[a] (distances in angstroms) and angles (in degrees) of X-ray structures and structures calculated using DFT//cc-pVTZ for N_4-Cu^{II} complexes.

Structure	CSD code	X	N_1-Cu	N_2-Cu	N_3-Cu	N_4-Cu	N_1-Cu-N_3	N_2-Cu-N_4
X-ray	HAFSUC[1]	$2x(BH_4)^{-1}$	2.021				179.97	
	IPEYUX[2]	$2x(C_7H_5O_2)^{-1}$,$2xH_2O$	1.969				180.00	
	IPEZAE[2]	$2x(C_{11}H_{13}O_2)^{-1}$,$2xH_2O$	2.011				180.00	
	AVERAGE		2.000				179.99	
	STD		0.028				0.01	
SVWN			2.004	1.99912	1.97796	1.999	161.414	159.75
BP86			2.05202	2.06054	2.03124	2.05821	161.862	161.996
B3LYP			2.05842	2.05725	2.05034	2.02959	162.14	162.106
CAMB3LYP			2.03622	2.03486	2.02905	2.00863	161.975	161.495
B3PW91			2.05283	2.04168	2.03514	2.01498	162.192	162.273
M06L			2.05248	2.05506	2.04747	2.02608	161.775	161.645
M06			2.0363	2.03354	2.02721	2.00474	159.775	161.31
M062X			2.06021	2.0599	2.05203	2.03106	159.882	160.653
M06HF			2.06544	2.06422	2.05425	2.03458	159.682	157.968
B97D			2.07885	2.07388	2.06757	2.04321	161.948	161.503
ωB97xD			2.03817	2.0369	2.03121	2.01056	161.798	160.17
PBE0			2.0349	2.03583	2.02862	2.0085	162.109	161.97
TPSSTPSS			2.04973	2.05182	2.02445	2.04357	162.206	162.711
		AVERAGE	2.04689	2.046508	2.034118	2.024055	161.4429	161.1962
		STD	0.017636	0.01847	0.020833	0.017218	0.93411	1.233475

Table S2: Comparison of critical bonds[a] (distances in angstroms) and angles (in degrees) of X-ray structures and structures calculated using DFT//cc-pVTZ for N_4-Cu^I complexes.

Structure	CSD code	X	N_1-Cu	N_2-Cu	N_3-Cu	N_4-Cu	N_1-Cu-N_3	N_2-Cu-N_4
SVWN			2.12067	2.06082	2.04474	2.01716	145.009	154.416
BP86			2.0935	2.05162	2.1477	2.24388	141.786	161.01
B3LYP			2.1458	2.09904	2.14451	2.23146	144.47	161.346
CAMB3LYP			2.1379	2.09195	2.11416	2.19254	145.733	159.08
B3PW91			2.14621	2.09803	2.10906	2.18359	147.062	157.978
M06L			2.08829	2.04616	2.29902	2.17632	136.905	166.55
M06			2.12793	2.0763	2.20941	2.12255	143.992	160.831
M062X			2.14398	2.20693	2.1992	2.15708	147.221	159.753
M06HF			2.17743	2.2066	2.19456	2.14719	149.12	159.539
B97D			2.13011	2.08505	2.1587	2.24266	140.633	164.489
ωB97xD			2.13652	2.08328	2.11796	2.22173	143.55	161.085
PBE0			2.14485	2.09642	2.1016	2.17481	147.128	157.81
TPSSTPSS			2.09865	2.05657	2.12852	2.21234	142.967	159.21
		AVERAGE	2.130134	2.096831	2.151472	2.178716	144.2751	160.23823
		STD	0.023906	0.04998	0.062825	0.058868	3.123795	2.8932959

Table S3: Comparison of critical bonds (distances in angstroms) and angles (in degrees) of X-ray structures and structures calculated using DFT//cc-pVTZ for N_3S_1-Cu^{II} complexes.

Structure	CSD code	X	N_1-Cu	N_2-Cu	N_4-Cu	S_3-Cu	N_2-Cu-N_4	N_1-Cu-S_3
SVWN			1.98242	1.99462	2.00612	2.24179	156.337	149.941
BP86			2.03634	2.06135	2.06945	2.30376	156.743	152.878
B3LYP			2.03439	2.06813	2.06465	2.32559	156.81	154.221
CAMB3LYP			2.0398	2.04245	2.01169	2.30442	156.567	153.649
B3PW91			2.04712	2.05056	2.01896	2.3021	157.092	153.862
M06L			2.05636	2.06442	2.03265	2.0916	156.145	153.246
M06			2.03038	2.03816	2.01318	2.31143	155.777	151.107
M062X			2.06334	2.06188	2.0308	2.37178	155.48	152.324
M06HF			2.06504	2.06655	2.02765	2.38531	154.447	150.551
B97D			2.0869	2.09468	2.05445	2.31827	155.155	153.684
ωB97xD			2.04265	2.03881	2.0145	2.30238	155.704	152.042
PBE0			2.04178	2.03911	2.01196	2.29986	157.081	153.503
TPSSTPSS			2.05846	2.05178	2.0292	2.30226	157.181	153.366
		AVERAGE	2.044998	2.051731	2.029635	2.313701	156.1938	152.6442
		STD	0.023483	0.022445	0.020082	0.033547	0.806965	1.308329

Table S4: Comparison of critical bonds (distances in angstroms) and angles (in degrees) of X-ray structures and structures calculated using DFT//cc-pVTZ for N_3S_1-Cu^I complexes.

Structure	CSD code	X	N_1-Cu	N_2-Cu	N_4-Cu	S_3-Cu	N_2-Cu-N_4	N_1-Cu-S_3
SVWN			2.02577	2.03849	2.08549	2.2622	144.527	142.602
BP86			2.13605	2.08303	2.18388	2.30254	143.968	147.09
B3LYP			2.15021	2.1046	2.20274	2.36857	146.161	148.04
CAMB3LYP			2.11177	2.0918	2.16207	2.37475	147.321	145.574
B3PW91			2.12891	2.08657	2.17864	2.33269	145.671	147.009
M06L			2.23282	2.16941	2.0715	2.30093	139.093	153.529
M06			2.17942	2.12225	2.08121	2.36082	144.963	148.13
M062X			2.19645	2.14952	2.13817	2.49343	149.362	146.706
M06HF			2.19457	2.16244	2.14257	2.50343	149.169	148.45
B97D			2.19723	2.16053	2.1109	2.3349	143.327	149.458
ωB97xD			2.18803	2.12268	2.08542	2.35085	144.775	147.638
PBE0			2.16886	2.1199	2.08346	2.33608	145.879	146.757
TPSSTPSS			2.16214	2.11597	2.08078	2.30915	144.298	145.762
		AVERAGE	2.159402	2.117476	2.123602	2.35618	145.2703	147.44192
		STD	0.049949	03.036016	0.044726	0.067625	2.525877	2.386839

Table S5: Comparison of critical bonds[a] (distances in angstroms) and angles (in degrees) of X-ray structures and structures calculated using DFT//cc-pVTZ for N_2S_2-Cu^{II} complexes.

Structure	CSD code	X	N_1-Cu	N_3-Cu	S_2-Cu	S_4-Cu	N_1-Cu-S_3	N_4-Cu-S_2
X-ray	ZUDSOG[3]	2x$(ClO_4)^-$	2.073(7)	2.073(7)	2.344(3)	2.344(3)	148.6(2)	178.6(2)
SVWN			2.01447	2.01447	2.25167	2.25167	159.434	159.41
BP86			2.08018	2.08018	2.31424	2.31424	159.335	160.238
B3LYP			2.07805	2.07805	2.32823	2.32823	159.504	160.025
CAMB3LYP			2.05124	2.05124	2.30627	2.30627	159.614	160.481
B3PW91			2.06017	2.06017	2.30892	2.30892	159.769	160.95
M06L			2.07442	2.07442	2.31836	2.31836	158.727	160.827
M06			2.04854	2.04854	2.32138	2.32138	158.732	159.842
M062X			2.06858	2.06858	2.36766	2.36766	158.789	160.268
M06HF			2.07119	2.07119	2.37516	2.37516	158.184	160.035
B97D			2.11015	2.11015	2.33186	2.33186	156.998	161.112
ωB97xD			2.0586	2.0586	2.30566	2.30566	158.803	161.217
PBE0			2.05084	2.05084	2.30526	2.30526	159.883	160.948
TPSSTPSS			2.0662	2.0662	2.31224	2.31224	159.977	160.419
		AVERAGE	2.064048	2.064048	2.318993	2.318993	159.0576	160.444
		STD	0.021208	0.021208	0.029103	0.029103	0.790207	0.52259

Table S6: Comparison of critical bonds (distances in angstroms) and angles (in degrees) of X-ray structures and structures calculated using DFT//cc-pVTZ for N_2S_2-Cu^I complexes.

Structure	CSD code	X	N_1-Cu	N_3-Cu	S_2-Cu	S_4-Cu	N_1-Cu-S_3	N_4-Cu-S_2
SVWN			2.09979	2.09979	2.27723	2.27723	146.418	155.573
BP86			2.23922	2.23922	2.30995	2.30995	145.989	159.588
B3LYP			2.25508	2.25508	2.35996	2.35996	145.642	162.274
CAMB3LYP			2.20501	2.20501	2.36052	2.36052	146.934	160.653
B3PW91			2.22563	2.22563	2.33407	2.33407	146.564	160.908
M06L			2.26241	2.26241	2.31526	2.31526	140.924	164.862
M06			2.21129	2.21129	2.35311	2.35311	142.026	163.445
M062X			2.19956	2.19956	2.48134	2.48134	145.938	163.245
M06HF			2.1957	2.1957	2.50558	2.50558	141.149	169.449
B97D			2.24295	2.24295	2.36107	2.36107	144.379	162.313
ωB97xD			2.25955	2.25955	2.32422	2.32422	140.965	165.617
PBE0			2.20822	2.20822	2.33911	2.33911	146.688	160.734
TPSSTPSS			2.20056	2.20056	2.32459	2.32459	146.697	158.273
		AVERAGE	2.215767	2.215767	2.347385	2.347385	144.6395	162.07185
		STD	0.040728	0.040728	0.062559	0.062559	2.344587	3.352721

Table S7: Comparison of critical bonds (distances in angstroms) and angles (in degrees) of X-ray structures and structures calculated using DFT//cc-pVTZ for NSSN-Cu^{II} complexes.

Structure	CSD code	X	N_1-Cu	S_2-Cu	N_4-Cu	S_3-Cu	N_1-Cu-S_3	N_2-Cu-S_4
SVWN			2.00793	2.2163	1.98378	2.23495	150.259	151.898
BP86			2.06692	2.806	2.04148	2.30337	150.879	153.154
B3LYP			2.06403	2.30996	2.03892	2.32748	151.293	154.275
CAMB3LYP			2.03849	2.28799	2.01424	2.30378	151.429	154.541
B3PW91			2.04671	2.28571	2.02271	2.30277	151.619	154.383
M06L			2.06459	2.29217	2.03778	2.30985	150.046	154.354
M06			2.04291	2.29119	2.01309	2.30638	148.908	153.518
M062X			2.02892	2.36891	2.05525	2.35872	149.484	155.518
M06HF			2.02571	2.38783	2.04813	2.37935	147.952	153.16
B97D			2.10004	2.29884	2.06377	2.32326	148.91	153.632
ωB97xD			2.01807	2.30276	2.04343	2.28625	150.578	153.989
PBE0			2.01438	2.29937	2.03828	2.28253	151.596	154.64
TPSSTPSS			2.0552	2.28278	2.03286	2.3015	151.365	153.87
		AVERAGE	2.044146	2.300339	2.33363	2.309245	150.3322	153.8786
		STD	0.024912	0.039938	0.020024	0.033774	1.154533	0.7893

Table S8: Comparison of critical bonds (distances in angstroms) and angles (in degrees) of X-ray structures and structures calculated using DFT//cc-pVTZ for NSSN-Cu^{I} complexes.

Structure	CSD code	X	N_1-Cu	S_2-Cu	N_4-Cu	S_3-Cu	N_1-Cu-S_3	N_2-Cu-S_4
SVWN			2.0716	2.21718	2.03085	2.27552	139.467	139.738
BP86			2.15556	2.28396	2.10659	2.34961	141.889	141.472
B3LYP			2.17409	2.32723	2.12458	2.40393	143.932	142.586
CAMB3LYP			2.14767	2.31109	2.09947	2.38418	143.438	142.19
B3PW91			2.1528	2.29811	2.10367	2.36677	143.005	142.317
M06L			2.17283	2.30072	2.10624	2.37203	143.728	140.792
M06			2.16214	2.32644	2.09085	2.36717	144.358	140.347
M062X			2.13218	2.45982	2.19515	2.42632	147.867	141.064
M06HF			2.13218	2.45982	2.19515	2.42632	147.867	141.064
B97D			2.12957	2.38386	2.18544	2.30699	142.828	141.218
ωB97xD			2.09527	2.37605	2.16167	2.30611	142.246	141.854
PBE0			2.09748	2.36325	2.14615	2.29531	142.322	142.935
TPSSTPSS			2.09682	2.34772	2.14036	2.28049	141.279	141.511
		AVERAGE	2.132704	2.343742	2.128872	2.352358	143.5745	141.47192
		STD	0.031663	0.067053	0.044508	0.053563	2.647294	0.8706514

Table S9: Comparison of critical bonds (distances in angstroms) and angles (in degrees) of X-ray structures and structures calculated using DFT//cc-pVTZ for N_1S_3-Cu^{II} complexes.

Structure	CSD code	X	N_1-Cu	S_2-Cu	S_3-Cu	S_4-Cu	N_1-Cu-S_3	S_2-Cu-S_4
SVWN			2.00867	2.20979	2.23859	2.20707	144.911	146.285
BP86			2.07476	2.28378	2.30388	2.27375	145.465	148.057
B3LYP			2.07553	2.30945	2.2945	2.32574	145.782	150.374
CAMB3LYP			2.04772	2.28472	2.30217	2.27038	146.094	150.799
B3PW91			2.05625	2.8266	2.30074	2.26999	146.554	150.085
M06L			2.07346	2.29155	2.31375	2.27765	144.285	149.947
M06			2.04849	2.28774	2.30868	2.7654	143.033	148.767
M062X			2.061	2.35425	2.33167	2.36708	144.563	152.088
M06HF			2.05366	2.38249	2.3753	2.3454	142.819	150.978
B97D			2.11753	2.30283	2.32531	2.28959	141.623	149.181
ωB97xD			2.05411	2.28432	2.3066	2.26832	144.924	150.184
PBE0			2.04716	2.27799	2.29682	2.26577	146.639	150.511
TPSSTPSS			2.06191	2.28046	2.27161	2.30115	146.332	148.871
		AVERAGE	2.060019	2.294772	2.304975	2.287572	144.848	149.7042
		STD	0.023413	0.038986	0.030251	0.038882	1.502448	1.422396

Table S10: Comparison of critical bonds (distances in angstroms) and angles (in degrees) of X-ray structures and structures calculated using DFT//cc-pVTZ for N_1S_3-Cu^I complexes.

Structure	CSD code	X	N_1-Cu	S_2-Cu	S_3-Cu	S_4-Cu	N_1-Cu-S_3	S_2-Cu-S_4
X-ray	SOZZAI[4]	$(ClO_4)^-$	2.061(5)	2.254(2)	2.277(2)	2.240(2)	114.2(2)	137.25(8)
SVWN			2.05731	2.23018	2.27241	2.21846	131.261	136.257
BP86			2.15773	2.28691	2.30911	2.3435	132.633	138.694
B3LYP			2.18922	2.36599	2.32539	2.38631	133.177	142.85
CAMB3LYP			2.15246	2.34125	2.30691	2.36741	133.229	141.952
B3PW91			2.15948	2.32533	2.29699	2.35527	133.659	140.82
M06L			2.18997	2.35405	2.28794	2.35786	128.507	144.789
M06			2.16232	2.34971	2.31084	2.3678	131.493	141.844
M062X			2.19956	2.47955	2.3936	2.45097	128.43	151.316
M06HF			2.18466	2.48629	2.46749	2.41686	128.508	153.917
B97D			2.20032	2.33925	2.30761	2.38066	129.314	141.514
ωB97xD			2.17744	2.34189	2.29819	2.36209	131.297	142.783
PBE0			2.15114	2.32161	2.2935	2.35268	133.397	141.18
TPSSTPSS			2.13624	2.30365	2.28484	2.34139	132.658	138.41
		AVERAGE	2.162912	2.348128	2.319602	2.361635	131.351	142.79431
		STD	0.036163	0.066646	0.051111	0.050722	1.933703	4.7136647

Table S11: Comparison of critical bonds (distances in angstroms) and angles (in degrees) of X-ray structures and structures calculated using DFT//cc-pVTZ for S_4-Cu^{II} complexes.

Structure	CSD code	X	S_1-Cu	S_2-Cu	S_3-Cu	S_4-Cu	S_1-Cu-S_3	S_2-Cu-S_4
X-ray	VIMDOK[5]	$2x(ClO_4)^-$	2.292(1)		2.312(1)		179.97	
	THCDCU[6]	$2x(ClO_4)^-$	2.297(1)		2.308(1)		179.97	
	AVERAGE		2.288		2.309		179.97	
	STD		0.009		0.001		0.00	
SVWN			2.23112	2.20009	2.2333	2.2091	140.194	140.626
BP86			2.30422	2.26909	2.2801	2.3012	141.315	141.784
B3LYP			2.32594	2.30295	2.28972	2.32298	142.111	143.86
CAMB3LYP			2.29936	2.29665	2.27805	2.26506	142.62	144.552
B3PW91			2.2991	2.29655	2.27653	2.26445	142.631	144.153
M06L			2.3094	2.31081	2.27277	2.28573	143.199	140.731
M06			2.30344	2.26992	2.30515	2.2842	141.686	139.826
M062X			2.35665	2.32563	2.35945	2.34394	145.895	141.514
M06HF			2.36935	2.34572	2.3745	2.36541	144.123	141.102
B97D			2.33295	2.30182	2.32817	2.2871	140.125	138.559
ωB97xD			2.29961	2.27749	2.29763	2.26476	142.043	143.624
PBE0			2.29337	2.27129	2.29101	2.25909	142.76	144.661
TPSSTPSS			2.29698	2.26544	2.30022	2.27593	141.913	143.173
		AVERAGE	2.309345	2.287188	2.298969	2.286842	142.355	142.1665
		STD	0.032295	0.034119	0.035844	0.038581	1.472946	1.891187

Table S12: Comparison of critical bonds (distances in angstroms) and angles (in degrees) of X-ray structures and structures calculated using DFT//cc-pVTZ for S_4-Cu^I complexes.

Structure	CSD code	X	S_1-Cu	S_2-Cu	S_3-Cu	S_4-Cu	S_1-Cu-S_3	S_2-Cu-S_4
SVWN			2.27564	2.22342	2.26696	2.22482	126.708	130.81
BP86			2.34771	2.29717	2.3011	2.35396	129.113	132.144
B3LYP			2.39342	2.3355	2.34483	2.39893	131.549	133.804
CAMB3LYP			2.36843	2.324	2.37369	2.3138	131.362	133.79
B3PW91			2.35819	2.31347	2.36275	2.30494	133.455	131.072
M06L			2.37525	2.31614	2.38783	2.30837	131.429	131.24
M06			2.37677	2.32933	2.38645	2.32078	132.039	130.382
M062X			2.4651	2.40585	2.4641	2.41988	135.038	133.847
M06HF			2.4607	2.42055	2.46719	2.43755	135.115	137.232
B97D			2.38655	2.32343	2.32021	2.39368	130.703	127.958
ωB97xD			2.36523	2.31897	2.31088	2.38176	133.178	129.862
PBE0			2.35832	2.30066	2.30963	2.35328	133.541	131.131
TPSSTPSS			2.34164	2.29729	2.29318	2.34639	129.42	131.951
		AVERAGE	2.374842	2.323522	2.352985	2.350626	131.7423	131.94023
		STD	0.046713	0.046799	0.059942	0.055116	2.313263	2.2249844

Table S13: Comparison of critical bonds[a] (distances in angstroms) and angles (in degrees) of X-ray structures and structures calculated using DFT//cc-pVTZ//PCM for N_4-Cu^{II} complexes.

Structure	CSD code	X	N_1-Cu	N_2-Cu	N_3-Cu	N_4-Cu	N_1-Cu-N_3	N_2-Cu-N_4
X-ray	HAFSUC[1]	$2x(BH_4)^{-1}$	2.021				179.97	
	IPEYUX[2]	$2x(C_7H_5O_2)^{-1}$,$2xH_2O$	1.969				180.00	
	IPEZAE[2]	$2x(C_{11}H_{13}O_2)^{-1}$,$2xH_2O$	2.011				180.00	
	AVERAGE		2.000				179.99	
	STD		0.028				0.01	
SVWN			1.99185	1.97	1.99444	1.98928	159.687	161.654
BP86			2.0522	2.04229	2.04851	2.02199	162.014	162.586
B3LYP			2.05193	2.04953	2.02282	2.04259	162.289	162.787
CAMB3LYP			2.03083	2.02289	2.00353	2.02841	162.157	162.395
B3PW91			2.0361	2.03385	2.00863	2.02789	162.308	162.83
M06L			2.04432	2.04786	2.01907	2.03938	161.869	162.745
M06			1.99927	2.02052	2.0289	2.02536	161.605	160.074
M062X			2.02699	2.04653	2.05416	2.05563	161.604	161.175
M06HF			2.06005	2.06079	2.03086	2.04909	160.659	159.762
B97D			2.07155	2.06562	2.03383	2.05753	162.117	162.714
ωB97xD			2.03282	2.03046	2.00511	2.02449	161.781	161.232
PBE0			2.02764	2.02979	2.02212	2.00294	162.642	162.258
TPSSTPSS			2.04059	2.04379	2.03493	2.01631	162.199	163.19
		AVERAGE	2.035857	2.035686	2.023608	2.0292992	161.7639	161.954
		STD	0.021366	0.022983	0.016895	0.0190549	0.758053	1.053783

Table S14: Comparison of critical bonds[a] (distances in angstroms) and angles (in degrees) of structures calculated using DFT//cc-pVTZ//PCM for N_4-Cu^I complexes.

Structure	CSD code	X	N_1-Cu	N_2-Cu	N_3-Cu	N_4-Cu	N_1-Cu-N_3	N_2-Cu-N_4
SVWN			2.12939	2.05148	2.04889	2.00711	143.392	155.801
BP86			2.255	2.15747	2.08026	2.04228	139.921	162.659
B3LYP			2.25046	2.15809	2.12572	2.0842	142.013	163.51
CAMB3LYP			2.19884	2.12687	2.11947	2.0838	144.355	160.374
B3PW91			2.19076	2.13355	2.11495	2.08857	145.693	159.171
M06L			2.30863	2.18704	2.07742	2.03938	134.998	168.289
M06			2.22778	2.1366	2.10806	2.06147	141.299	163.094
M062X			2.19463	2.15336	2.14476	2.20638	146.371	160.77
M06HF			2.17426	2.20407	2.14659	2.19298	148.289	160.632
B97D			2.11349	2.07178	2.17069	2.26278	137.735	167.008
ωB97xD			2.10918	2.06925	2.1351	2.24822	140.752	163.631
PBE0			2.08932	2.1351	2.17861	2.1053	158.829	146.036
TPSSTPSS			2.0444	2.08246	2.22649	2.13986	161.11	140.781
		AVERAGE	2.175857	2.12824	2.129001	2.120179	144.9813	159.3658
		STD	0.072602	0.045053	0.045141	0.07967	7.265458	7.53736

Table S15: Comparison of critical bonds (distances in angstroms) and angles (in degrees) of X-ray structures and structures calculated using DFT//cc-pVTZ//PCM for N_3S_1-Cu^{II} complexes.

Structure	CSD code	X	N_1-Cu	N_2-Cu	N_4-Cu	S_3-Cu	N_2-Cu-N_4	N_1-Cu-S_3
SVWN			1.97547	1.98429	1.99598	2.24197	156.308	149.979
BP86			2.04201	2.0283	2.05075	2.30927	156.279	151.628
B3LYP			2.04723	2.02719	2.05156	2.33828	156.799	153.036
CAMB3LYP			2.02359	2.02703	2.00556	2.31762	156.459	152.15
B3PW91			2.02963	2.03403	2.01235	2.31302	156.895	152.375
M06L			2.04022	2.05052	2.02495	2.32042	156.101	152.937
M06			2.0177	2.02665	2.00515	2.31975	155.978	150.765
M062X			2.05147	2.04706	2.02388	2.39132	156.583	151.386
M06HF			2.0542	2.05345	2.02109	2.40651	156.001	150.039
B97D			2.06337	2.07336	2.04696	2.32973	155.554	151.819
ωB97xD			2.02342	2.02888	2.00859	2.31546	155.914	150.915
PBE0			2.02572	2.02232	2.00595	2.312	157.049	151.778
TPSSTPSS			2.04113	2.03408	2.02178	2.3095	156.847	152.071
		AVERAGE	2.033474	2.033628	2.021119	2.3249885	156.3667	151.606
		STD	0.21304	0.01997	0.017733	0.0383598	0.434076	0.933612

Table S16: Comparison of critical bonds (distances in angstroms) and angles (in degrees) of X-ray structures and structures calculated using DFT//cc-pVTZ//PCM for N_3S_1-Cu^I complexes.

Structure	CSD code	X	N_1-Cu	N_2-Cu	N_4-Cu	S_3-Cu	N_2-Cu-N_4	N_1-Cu-S_3
SVWN			2.02274	2.03075	2.07725	2.2648	143.314	143.298
BP86			2.08277	2.12186	2.17156	2.31119	143.275	147.491
B3LYP			2.11732	2.11372	2.17331	2.41801	147.923	146.438
CAMB3LYP			2.0875	2.09684	2.14006	2.41773	148.723	144.371
B3PW91			2.08974	2.10853	2.16034	2.358	146.082	146.659
M06L			2.07285	2.14587	2.21561	2.31815	139.262	152.968
M06			2.09857	2.16113	2.08445	2.39259	145.815	147.073
M062X			2.17683	2.12539	2.1412	2.53924	151.833	144.267
M06HF			2.18317	2.14691	2.14011	2.53097	149.753	147.746
B97D			2.1898	2.15029	2.10856	2.34652	142.399	150.599
ωB97xD			2.10302	2.17169	2.08641	2.38039	145.02	147.449
PBE0			2.15337	2.10106	2.08582	2.36057	146.318	146.421
TPSSTPSS			2.1517	2.10405	2.07997	2.31773	143.616	146.308
		AVERAGE	2.117645	2.121392	2.12805	2.381222	145.641	147.0068
		STD	0.047985	0.035047	0.042791	0.07788	3.250327	2.46367

Table S17: Comparison of critical bonds[a] (distances in angstroms) and angles (in degrees) of X-ray structures and structures calculated using DFT//cc-pVTZ//PCM for N_2S_2-Cu^{II} complexes.

Structure	CSD code	X	N_1-Cu	N_3-Cu	S_2-Cu	S_4-Cu	N_1-Cu-S_3	N_4-Cu-S_2
X-ray	ZUDSOG[3]	2x$(ClO_4)^-$	2.073(7)	2.073(7)	2.344(3)	2.344(3)	148.6(2)	178.6(2)
SVWN			1.99727	1.99727	2.25233	2.25233	157.938	159.386
BP86			2.06203	2.06203	2.31983	2.31983	158.115	161.011
B3LYP			2.06134	2.06134	2.34208	2.34208	158.289	161.439
CAMB3LYP			2.0373	2.0373	2.31853	2.31853	158.609	161.589
B3PW91			2.04411	2.04411	2.317	2.317	159.022	161.447
M06L			2.05595	2.05595	2.32405	2.32405	158.74	160.264
M06			2.0341	2.0341	2.3273	2.3273	157.997	160.591
M062X			2.05415	2.05415	2.37988	2.37988	157.788	161.019
M06HF			2.05801	2.05801	2.38953	2.38953	156.074	162.808
B97D			2.09335	2.09335	2.34028	2.34028	155.63	161.931
ωB97xD			2.04376	2.04376	2.31596	2.31596	157.748	162.283
PBE0			2.03454	2.03454	2.31192	2.31192	159.514	161.195
TPSSTPSS			2.04629	2.04629	2.3134	2.3134	159.417	159.941
		AVERAGE	2.047862	2.047862	2.327084	2.3270838	158.0678	161.1465
		STD	0.020996	0.020996	0.032168	0.0321676	1.100307	0.907925

Table S18: Comparison of critical bonds (distances in angstroms) and angles (in degrees) of X-ray structures and structures calculated using DFT//cc-pVTZ//PCM for N_2S_2-Cu^I complexes.

Structure	CSD code	X	N_1-Cu	N_4-Cu	S_2-Cu	S_3-Cu	N_1-Cu-S_3	N_4-Cu-S_2
SVWN			2.08409	2.08409	2.29833	2.28933	145.51	156.697
BP86			2.19235	2.19235	2.34073	2.34073	145.916	159.264
B3LYP			2.18384	2.18384	2.42355	2.42355	148.186	160.309
CAMB3LYP			2.14237	2.14237	2.4227	2.4227	149.247	159.039
B3PW91			2.17224	2.17224	2.37636	2.37636	147.565	159.916
M06L			2.20493	2.20493	2.34857	2.34857	140.12	163.935
M06			2.16304	2.16304	2.39035	2.39035	144.478	161.327
M062X			2.16512	2.16512	2.52825	2.52825	145.13	164.883
M06HF			2.17964	2.17964	2.53176	2.53176	139.886	171.544
B97D			2.1971	2.1971	2.39634	2.39634	145.333	161.413
ωB97xD			2.19751	2.19751	2.3712	2.3712	142.355	164.562
PBE0			2.16203	2.16203	2.37608	2.37608	147.539	159.984
TPSSTPSS			2.14808	2.14808	2.36265	2.36265	147.509	156.701
		AVERAGE	2.168642	2.168642	2.397452	2.397452	145.2903	161.5057
		STD	0.03068	0.03068	0.064897	0.064897	2.84031	3.833556

Table S19: Comparison of critical bonds (distances in angstroms) and angles (in degrees) of X-ray structures and structures calculated using DFT//cc-pVTZ//PCM for NSSN-Cu^{II} complexes.

Structure	CSD code	X	N_1-Cu	S_2-Cu	S_3-Cu	N_4-Cu	N_1-Cu-S_3	N_2-Cu-S_4
SVWN			1.99799	2.21447	1.97304	2.22925	149.949	150.904
BP86			2.05347	2.28174	2.02934	2.30319	150.902	151.148
B3LYP			2.05033	2.31113	2.02635	2.33356	151.602	152.05
CAMB3LYP			2.02714	2.29192	2.00209	2.30925	151.257	153.036
B3PW91			2.03533	2.28753	2.01046	2.30619	151.575	152.907
M06L			2.05437	2.29341	2.02531	2.31106	150.081	153.165
M06			2.03533	2.29353	2.00024	2.30599	148.294	153.241
M062X			2.04421	2.36857	2.01782	2.3791	149.726	153.966
M06HF			2.04093	2.39629	2.01378	2.39509	146.803	154.569
B97D			2.04859	2.32007	2.0901	2.29871	147.678	152.847
ωB97xD			2.03273	2.29073	2.00509	2.30718	150.316	152.863
PBE0			2.00245	2.30337	2.02764	2.28657	153.387	151.531
TPSSTPSS			2.02099	2.30265	2.04332	2.28105	151.4	151.998
		AVERAGE	2.03394	2.304262	2.020352	2.3112454	150.2285	152.6327
		STD	0.017408	0.041439	0.026229	0.0398765	1.72985	1.025297

Table S20: Comparison of critical bonds (distances in angstroms) and angles (in degrees) of X-ray structures and structures calculated using DFT//cc-pVTZ//PCM for NSSN-Cu^{I} complexes.

Structure	CSD code	X	N_1-Cu	N_2-Cu	S_3-Cu	S_4-Cu	N_1-Cu-S_3	N_2-Cu-S_4
SVWN			2.06268	2.21512	2.02391	2.2775	139.384	139.358
BP86			2.14222	2.28075	2.09839	2.35693	142.076	140.964
B3LYP			2.15772	2.32086	2.11707	2.42187	144.621	141.671
CAMB3LYP			2.13404	2.30694	2.09243	2.39849	143.962	141.515
B3PW91			2.13959	2.29492	2.09581	2.3775	143.308	141.824
M06L			2.15316	2.29109	2.10143	2.392	141.807	142.535
M06			2.1491	2.32523	2.08338	2.37489	140.443	144.088
M062X			2.12366	2.47079	2.18465	2.42739	141.007	148.071
M06HF			2.13089	2.48561	2.17847	2.45467	138.805	153.014
B97D			2.1215	2.39485	2.1726	2.30211	141.631	142.161
ωB97xD			2.08998	2.39455	2.14149	2.30066	140.778	143.073
PBE0			2.08955	2.37347	2.13347	2.29211	141.842	143.166
TPSSTPSS			2.08871	2.3555	2.12755	2.27694	140.724	141.661
		AVERAGE	2.121754	2.346898	2.119281	2.357928	141.5683	143.3155
		STD	0.028597	0.073507	0.042786	0.059164	161.2325	3.412591

Table S21: Comparison of critical bonds (distances in angstroms) and angles (in degrees) of X-ray structures and structures calculated using DFT//cc-pVTZ//PCM for N_1S_3-Cu^{II} complexes.

Structure	CSD code	X	N_1-Cu	S_2-Cu	S_3-Cu	S_4-Cu	N_1-Cu-S_3	S_2-Cu-S_4
SVWN			1.99853	2.23413	2.20813	2.20508	143.384	147.137
BP86			2.05876	2.28051	2.27275	2.30241	144.59	147.413
B3LYP			2.05799	2.30854	2.29548	2.32795	145.002	149.28
CAMB3LYP			2.03403	2.30388	2.28694	2.27126	145.106	150.217
B3PW91			2.04058	2.302	2.28269	2.27106	145.779	149.292
M06L			2.06245	2.29421	2.27521	2.31408	142.038	150.81
M06			2.03791	2.29149	2.27436	2.30823	141.235	150.032
M062X			2.05051	2.36869	2.36746	2.33683	143.089	152.246
M06HF			2.04604	2.39922	2.38015	2.35048	139.859	153.389
B97D			2.10661	2.30546	2.28662	2.32545	139.155	150.369
ωB97xD			2.0437	2.28942	2.30205	2.26943	143.775	150.377
PBE0			2.03505	2.28082	2.26616	2.29752	145.504	150.481
TPSSTPSS			2.04627	2.27772	2.27173	2.30126	145.614	148.063
		AVERAGE	2.047548	2.302776	2.289979	2.2985415	143.3946	149.9312
		STD	0.02295	0.039599	0.041826	0.036367	2.134324	1.694861

Table S22: Comparison of critical bonds (distances in angstroms) and angles (in degrees) of X-ray structures and structures calculated using DFT//cc-pVTZ//PCM for N_1S_3-Cu^I complexes.

Structure	CSD code	X	N_1-Cu	S_2-Cu	S_3-Cu	S_4-Cu	N_1-Cu-S_3	S_2-Cu-S_4
X-ray	SOZZAI[4]	$(ClO_4)^-$	2.061(5)	2.254(2)	2.277(2)	2.240(2)	114.2(2)	137.25(8)
SVWN			2.04601	2.22927	2.27158	2.21875	130.507	136.463
BP86			2.13817	2.30767	2.34511	2.29004	131.96	138.902
B3LYP			2.16608	2.33166	2.36163	2.3941	132.968	142.371
CAMB3LYP			2.13431	2.33794	2.31211	2.37499	133.048	141.79
B3PW91			2.13946	2.32272	2.361	2.30148	133.262	140.794
M06L			2.16397	2.34896	2.36658	2.29283	128.572	144.367
M06			2.14317	2.34751	2.37224	2.31454	131.095	141.913
M062X			2.1813	2.47467	2.40158	2.46135	128.64	150.822
M06HF			2.17342	2.48855	2.47787	2.424	127.627	154.7
B97D			2.18246	2.33716	2.31135	2.38557	128.596	141.929
ωB97xD			2.15625	2.33906	2.30366	2.37002	131.155	142.696
PBE0			2.13228	2.31897	2.35835	2.29731	132.966	141.181
TPSSTPSS			2.11957	2.30247	2.34281	2.28755	132.047	138.53
		AVERAGE	2.144342	2.345124	2.352759	2.339425	130.9572	142.8045
		STD	0.034257	0.065361	0.049076	0.065065	1.920343	4.734555

Table S23: Comparison of critical bonds (distances in angstroms) and angles (in degrees) of X-ray structures and structures calculated using DFT//cc-pVTZ//PCM for S_4-Cu^{II} complexes.

Structure	CSD code	X	S_1-Cu	S_2-Cu	S_3-Cu	S_4-Cu	S_1-Cu-S_3	S_2-Cu-S_4
X-ray	VIMDOK[5]	$2x(ClO_4)^-$	2.292(1)		2.312(1)		179.97	
	THCDCU[6]	$2x(ClO_4)^-$	2.297(1)		2.308(1)		179.97	
	AVERAGE		2.288		2.309		179.97	
	STD		0.009		0.001		0.00	
SVWN			2.22701	2.22805	2.19491	2.20361	138.262	140.101
BP86			2.29803	2.27512	2.26482	2.29894	140.924	139.423
B3LYP			2.3204	2.29899	2.28793	2.32558	142.192	140.556
CAMB3LYP			2.29622	2.27554	2.29957	2.26427	142.683	141.34
B3PW91			2.29467	2.27304	2.29767	2.26243	142.571	141.09
M06L			2.30674	2.28234	2.30969	2.26985	140.412	140.662
M06			2.3012	2.28143	2.30207	2.26548	139.587	139.642
M062X			2.36374	2.33002	2.36161	2.3486	142.742	141.316
M06HF			2.37456	2.35077	2.37986	2.37221	142.794	140.702
B97D			2.32951	2.2977	2.2825	2.32638	138.587	137.779
ωB97xD			2.29735	2.27583	2.26445	2.30002	141.981	140.968
PBE0			2.2925	2.25792	2.29033	2.26885	141.677	142.78
TPSSTPSS			2.29581	2.26216	2.29424	2.27152	140.352	141.682
		AVERAGE	2.307518	2.283762	2.294565	2.2905954	141.1357	140.6185
		STD	0.034725	0.0299	0.042988	0.0426426	1.525247	1.172793

Table S24: Comparison of critical bonds (distances in angstroms) and angles (in degrees) of X-ray structures and structures calculated using DFT//cc-pVTZ//PCM for S_4-Cu^I complexes.

Structure	CSD code	X	S_1-Cu	S_2-Cu	S_3-Cu	S_4-Cu	S_1-Cu-S_3	S_2-Cu-S_4
SVWN			2.27395	2.22179	2.26449	2.22269	126.223	130.388
BP86			2.3465	2.29804	2.35197	2.29577	128.56	131.815
B3LYP			2.39234	2.34111	2.39938	2.33502	133.809	130.675
CAMB3LYP			2.36759	2.32069	2.37474	2.31348	133.761	130.632
B3PW91			2.35682	2.31007	2.36242	2.304	133.238	130.383
M06L			2.38901	2.30876	2.37173	2.31168	131.539	130.218
M06			2.37469	2.32374	2.38705	2.31956	132.049	129.414
M062X			2.46397	2.4037	2.46733	2.41786	134.003	134.069
M06HF			2.46064	2.42293	2.4715	2.43722	134.959	136.737
B97D			2.38454	2.32	2.31979	2.39403	130.674	127.087
ωB97xD			2.36419	2.31538	2.31119	2.38351	133.145	128.989
PBE0			2.35842	2.29988	2.30635	2.35204	130.499	133.326
TPSSTPSS			2.34459	2.29172	2.29423	2.33966	128.79	131.588
		AVERAGE	2.375173	2.32137	2.360167	2.340502	131.6345	131.1785
		STD	0.046874	0.047729	0.060118	0.055073	2.480482	2.350396

Table S25: Comparison of critical bonds[a] (distances in angstroms) and angles (in degrees) of X-ray structures and structures calculated using DFT//cc-pVTZ//SMD for N_4-Cu^{II} complexes.

Structure	CSD code	X	N_1-Cu	N_2-Cu	N_3-Cu	N_4-Cu	N_1-Cu-N_3	N_2-Cu-N_4
X-ray	HAFSUC[1]	$2x(BH_4)^{-1}$	2.021				179.97	
	IPEYUX[2]	$2x(C_7H_5O_2)^{-1}$,$2xH_2O$	1.969				180.00	
	IPEZAE[2]	$2x(C_{11}H_{13}O_2)^{-1}$,$2xH_2O$	2.011				180.00	
	AVERAGE		2.000				179.99	
	STD		0.028				0.01	
SVWN			1.9945	1.97031	1.99358	1.99961	161.074	162.235
BP86			2.05608	2.05873	2.04529	2.02269	161.583	163.583
B3LYP			2.06026	2.06431	2.04726	2.02616	160.961	164.732
CAMB3LYP			2.03673	2.04605	2.00756	2.0278	161.275	164.485
B3PW91			2.04136	2.04944	2.0315	2.01198	161.394	164.768
M06L			2.05849	2.0553	2.04257	2.02185	160.65	164.54
M06			2.03388	2.04201	2.02652	2.00085	161.328	161.821
M062X			2.07756	2.07633	2.03651	2.05597	158.376	164.982
M06HF			2.0866	2.8555	2.04477	2.06387	157.956	163.995
B97D			2.08047	2.07703	2.03547	2.06064	160.933	164.234
ωB97xD			2.03959	2.04808	2.00921	2.03056	161.102	163.263
PBE0			2.03552	2.04511	2.00703	2.02643	161.34	164.683
TPSSTPSS			2.05253	2.04797	2.03727	2.01827	161.573	164.775
		AVERAGE	2.050275	2.051248	2.028042	2.028206	161.7342	164.0251
		STD	0.023462	0.026945	0.017068	0.01982	1.126592	0.967627

Table S26: Comparison of critical bonds[a] (distances in angstroms) and angles (in degrees) of X-ray structures and structures calculated using DFT//cc-pVTZ//SMD for N_4-Cu^I complexes.

Structure	CSD code	X	N_1-Cu	N_2-Cu	N_3-Cu	N_4-Cu	N_1-Cu-N_3	N_2-Cu-N_4
SVWN			2.1332	2.05342	2.05602	2.01241	142.69	155.891
BP86			2.29368	2.07468	2.17436	2.03726	138.236	163.338
B3LYP			2.29738	2.12367	2.1764	2.07811	139.38	165.082
CAMB3LYP			2.14754	2.09687	2.1989	2.11752	144.649	159.284
B3PW91			2.15041	2.10013	2.19378	2.11525	145.587	158.442
M06L			2.06803	2.02715	2.38948	2.22305	132.712	170.071
M06			2.10175	2.05421	2.15388	2.26906	138.568	164.363
M062X			2.15333	2.22687	2.15926	2.21249	145.262	160.745
M06HF			2.1835	2.22195	2.15797	2.21205	145.592	161.715
B97D			2.18323	2.30779	2.06821	2.11501	135.526	168.194
ωB97xD			2.15267	2.28039	2.06761	2.10838	138.685	164.605
PBE0			2.10803	2.18627	2.09793	2.14916	145.415	158.542
TPSSTPSS			2.15238	2.25212	2.0427	2.08126	139.23	161.669
		AVERAGE	2.163472	2.154271	2.148962	2.133155	140.8871	162.457
		STD	0.064063	0.091888	0.087545	0.073554	4.105493	3.878242

Table S27: Comparison of critical bonds (distances in angstroms) and angles (in degrees) of X-ray structures and structures calculated using DFT//cc-pVTZ//SMD for N_3S_1-Cu^{II} complexes.

Structure	CSD code	X	N_1-Cu	N_2-Cu	N_4-Cu	S_3-Cu	N_2-Cu-N_4	N_1-Cu-S_3
SVWN			1.9889	1.97435	1.98414	2.27873	157.947	149.162
BP86			2.04187	2.04389	2.04474	2.3455	159.656	149.599
B3LYP			2.04761	2.04513	2.04252	2.38693	160.496	149.313
CAMB3LYP			2.02305	2.02256	2.02264	2.36777	160.25	149.41
B3PW91			2.03001	2.02851	2.02863	2.35705	160.839	149.511
M06L			2.05052	2.04029	2.02493	2.32029	156.069	152.951
M06			2.00783	2.01655	2.02132	2.37301	158.756	147.83
M062X			2.05146	2.04709	2.02392	2.39111	156.564	151.388
M06HF			2.05411	2.05332	2.02108	2.40661	156.046	149.978
B97D			2.06489	2.06336	2.06047	2.37609	158.485	148.711
ωB97xD			2.01909	2.02477	2.02657	2.36996	159.1	148.461
PBE0			2.02584	2.02249	2.00585	2.31186	157.025	151.828
TPSSTPSS			2.0412	2.03407	2.02177	2.30946	156.833	152.078
		AVERAGE	2.034337	2.032029	2.025275	2.353413	158.3128	150.0169
		STD	0.020338	0.02122	0.017654	0.036334	1.635287	1.492354

Table S28: Comparison of critical bonds (distances in angstroms) and angles (in degrees) of X-ray structures and structures calculated using DFT//cc-pVTZ//SMD for N_3S_1-Cu^I complexes.

Structure	CSD code	X	N_1-Cu	N_2-Cu	N_4-Cu	S_3-Cu	N_2-Cu-N_4	N_1-Cu-S_3
SVWN			2.02186	2.04074	2.09117	2.25897	141.451	145.452
BP86			2.07783	2.13617	2.19183	2.30179	141.141	148.981
B3LYP			2.14478	2.10236	2.2088	2.39302	143.527	150.297
CAMB3LYP			2.10408	2.16815	2.08964	2.40008	144.919	147.781
B3PW91			2.12446	2.18469	2.08489	2.34489	143.241	148.995
M06L			2.15327	2.22374	2.07705	2.32356	138.811	153.053
M06			2.1195	2.18519	2.07787	2.37527	142.444	150.057
M062X			2.11498	2.17468	2.16632	2.6175	156.161	140.723
M06HF			2.20387	2.1581	2.14545	2.56213	144.313	153.163
B97D			2.20304	2.1588	2.1062	2.34763	140.348	151.788
ωB97xD			2.19448	2.11817	2.08339	2.3678	142.078	149.884
PBE0			2.17638	2.11539	2.08183	2.34972	143.335	148.938
TPSSTPSS			2.17534	2.11862	2.07387	2.30592	140.997	148.423
		AVERAGE	2.139528	2.144985	2.113716	2.380637	143.2897	149.0412
		STD	0.051284	0.044917	0.045612	0.097251	4.053326	3.137973

Table S29: Comparison of critical bonds[a] (distances in angstroms) and angles (in degrees) of X-ray structures and structures calculated using DFT//cc-pVTZ//SMD for N_2S_2-Cu^{II} complexes.

Structure	CSD code	X	N_1-Cu	N_3-Cu	S_2-Cu	S_4-Cu	N_1-Cu-S_3	N_4-Cu-S_2
X-ray	ZUDSOG[3]	2x$(ClO_4)^-$	2.073(7)	2.073(7)	2.344(3)	2.344(3)	148.6(2)	178.6(2)
SVWN			1.99897	1.99897	2.28075	2.28075	156.79	164.811
BP86			2.05928	2.05928	2.35	2.35	156.739	164.138
B3LYP			2.0599	2.0599	2.37792	2.37792	156.62	164.179
CAMB3LYP			2.03667	2.03667	2.35401	2.35401	156.531	164.895
B3PW91			2.04313	2.04313	2.35131	2.35131	156.856	164.892
M06L			2.05548	2.05548	2.32276	2.32276	158.371	160.038
M06			2.02738	2.02738	2.35647	2.35647	157.46	163.724
M062X			2.05409	2.05409	2.38001	2.38001	157.802	161.003
M06HF			2.05796	2.05796	2.38963	2.38963	156.091	162.8
B97D			2.08395	2.08395	2.38014	2.38014	155.767	164.506
ωB97xD			2.04203	2.04203	2.35443	2.35443	156.28	164.904
PBE0			2.03434	2.03434	2.31211	2.31211	159.562	161.119
TPSSTPSS			2.04616	2.04616	2.31348	2.31348	159.451	159.919
		AVERAGE	2.016103	2.046103	2.348617	2.348617	157.2554	163.1483
		STD	0.019441	0.019441	0.031278	03.031278	1.170802	1.86257

Table S30: Comparison of critical bonds (distances in angstroms) and angles (in degrees) of X-ray structures and structures calculated using DFT//cc-pVTZ//SMD for N_2S_2-Cu^I complexes.

Structure	CSD code	X	N_1-Cu	N_3-Cu	S_2-Cu	S_4-Cu	N_1-Cu-S_3	N_4-Cu-S_2
SVWN			2.06139	2.06139	2.3225	2.3225	145.24	157.352
BP86			1.98881	1.98881	2.79127	2.79126	167.133	151.27
B3LYP			2.00079	2.00079	2.88005	2.88005	170.828	148.869
CAMB3LYP			1.98933	1.98933	2.8263	2.8263	169.039	150.73
B3PW91			2.03404	2.03404	2.64763	2.64763	161.622	153.69
M06L			1.99863	1.99863	2.81053	2.81053	170.817	148.218
M06			1.99804	1.99804	2.74095	2.74094	165.553	151.665
M062X			2.10718	2.10718	2.65341	2.65341	158.681	155.851
M06HF			2.16618	2.16618	2.58178	2.58178	171.939	140.633
B97D			1.99343	1.99343	2.92377	2.92377	171.241	148.075
ωB97xD			1.99169	1.99169	2.8451	2.8451	169.557	150.626
PBE0			2.08447	2.08447	2.5154	2.5154	155.64	155.386
TPSSTPSS			2.03485	2.03485	2.59405	2.59405	161.358	150.548
		AVERAGE	2.034525	2.034525	2.702517	2.702517	164.5114	150.9933
		STD	0.053524	0.053524	0.163692	0.163691	7.526397	4.109986

Table S31: Comparison of critical bonds (distances in angstroms) and angles (in degrees) of X-ray structures and structures calculated using DFT//cc-pVTZ//SMD for NSSN-Cu^{II} complexes.

Structure	CSD code	X	N_1-Cu	S_2-Cu	N_4-Cu	S_3-Cu	N_1-Cu-S_3	N_2-Cu-S_4
SVWN			1.99769	2.23735	1.96909	2.25311	149.88	155.623
BP86			2.05269	2.30904	2.02332	2.32372	149.539	156.013
B3LYP			2.05139	2.34049	2.01871	2.36045	149.314	157.727
CAMB3LYP			2.0278	2.32261	1.99553	2.32261	149.718	157.853
B3PW91			2.03483	2.31642	2.00358	2.33094	149.823	157.711
M06L			2.02529	2.31097	2.05433	2.29344	150.1	153.157
M06			2.02914	2.32227	1.99596	2.32914	148.405	155.756
M062X			2.01792	2.37909	2.0443	2.36865	149.789	153.992
M06HF			2.01255	2.39622	2.03896	2.3926	146.177	154.667
B97D			2.08541	2.32944	2.04693	2.34793	147.317	156.579
ωB97xD			2.03271	2.32447	1.99969	2.33334	149.227	157.251
PBE0			2.02762	2.28671	2.00243	2.30339	151.537	153.392
TPSSTPSS			2.0211	2.30268	2.04315	2.28098	151.957	151.418
		AVERAGE	2.032011	2.321366	2.018152	2.326177	149.4448	155.4722
		STD	0.020806	0.03749	0.024988	0.036096	1.467702	1.951581

Table S32: Comparison of critical bonds (distances in angstroms) and angles (in degrees) of X-ray structures and structures calculated using DFT//cc-pVTZ//SMD for NSSN-Cu^{I} complexes.

Structure	CSD code	X	N_1-Cu	S_2-Cu	N_4-Cu	S_3-Cu	N_1-Cu-S_3	N_2-Cu-S_4
SVWN			2.06292	2.2201	2.02652	2.28615	139.879	139.274
BP86			2.13193	2.28523	2.10936	2.375	143.497	140.134
B3LYP			2.14049	2.30747	2.13625	2.48601	149.108	137.44
CAMB3LYP			2.12434	2.30758	2.10306	2.42711	146.165	139.69
B3PW91			2.12979	2.29846	2.10643	2.3984	145.044	140.594
M06L			2.12983	2.2687	2.12036	2.47106	147.466	137.423
M06			2.14944	2.3419	2.08313	2.38035	144.457	140.447
M062X			2.12434	2.46717	2.20842	2.47508	153.83	135.911
M06HF			2.13846	2.49269	2.20128	2.5044	160.473	132.262
B97D			2.14106	2.4758	2.15184	2.27804	147.522	136.607
ωB97xD			2.1268	2.29428	2.10133	2.43931	146.477	137.608
PBE0			2.12544	2.29636	2.09901	2.39353	144.97	140.581
TPSSTPSS			2.12048	2.28299	2.09777	2.37235	143.154	140.054
		AVERAGE	2.126563	2.333748	2.118828	2.406676	147.0802	138.3096
		STD	0.020034	0.083649	0.046119	0.068129	5.00904	2.343785

Table S33: Comparison of critical bonds (distances in angstroms) and angles (in degrees) of X-ray structures and structures calculated using DFT//cc-pVTZ//SMD for N_1S_3-Cu^{II} complexes.

Structure	CSD code	X	N_1-Cu	S_2-Cu	S_3-Cu	S_4-Cu	N_1-Cu-S_3	S_2-Cu-S_4
SVWN			1.99465	2.22426	2.24271	2.21234	147.198	144.241
BP86			2.05115	2.29921	2.28158	2.31815	147.584	146.292
B3LYP			2.05743	2.33583	2.32526	2.3753	142.264	158.106
CAMB3LYP			2.0349	2.34681	2.31678	2.30237	142.719	160.718
B3PW91			2.04384	2.30983	2.2992	2.34401	142.943	159.894
M06L			2.06202	2.29424	2.27531	2.31418	142.028	150.83
M06			2.03564	2.31116	2.30331	2.34724	141.23	156.449
M062X			2.05044	2.36868	2.33681	2.36755	143.093	152.229
M06HF			2.04595	2.39912	2.38002	2.35056	139.922	153.354
B97D			2.11596	2.33842	2.31925	2.36814	135.018	160.022
ωB97xD			2.04233	2.31389	2.3476	2.30099	142.257	160.617
PBE0			2.03519	2.281	2.29734	2.26617	145.471	150.533
TPSSTPSS			2.0462	2.27782	2.30118	2.27184	145.617	148.091
		AVERAGE	2.047362	2.315405	2.309719	2.318372	142.8726	153.952
		STD	0.025277	0.042467	0.033209	0.045886	3.160192	5.53268

Table S34: Comparison of critical bonds (distances in angstroms) and angles (in degrees) of X-ray structures and structures calculated using DFT//cc-pVTZ//SMD for N_1S_3-Cu^I complexes.

Structure	CSD code	X	N_1-Cu	S_2-Cu	S_3-Cu	S_4-Cu	N_1-Cu-S_3	S_2-Cu-S_4
X-ray	SOZZAI[4]	$(ClO_4)^-$	2.061(5)	2.254(2)	2.277(2)	2.240(2)	114.2(2)	137.25(8)
SVWN			2.0423	2.23098	2.27217	2.222	130.497	135.963
BP86			2.13436	2.30903	2.29744	2.35341	133.812	137.024
B3LYP			2.16162	2.3646	2.34902	2.41189	136.455	139.604
CAMB3LYP			2.12839	2.38459	2.33799	2.32431	135.818	138.996
B3PW91			2.13435	2.36958	2.32218	2.31107	135.842	137.988
M06L			2.1502	2.33218	2.39493	2.31405	133.473	139.831
M06			2.13494	2.34245	2.38642	2.32563	133.533	139.352
M062X			2.20351	2.55735	2.48568	2.42767	119.899	161.867
M06HF			2.19574	2.5532	2.51324	2.46301	118.041	165.926
B97D			2.17785	2.33449	2.39861	2.32513	131.481	139.085
ωB97xD			2.14481	2.33514	2.3839	2.31351	133.742	139.622
PBE0			2.12744	2.31864	2.36539	2.30733	135.721	138.133
TPSSTPSS			2.11644	2.30303	2.35047	2.29526	133.978	136.567
		AVERAGE	2.142458	2.364251	2.373649	2.338022	131.7148	142.3045
		STD	0.038801	0.089144	0.064819	0.060762	5.687637	9.316457

Table S35: Comparison of critical bonds (distances in angstroms) and angles (in degrees) of X-ray structures and structures calculated using DFT//cc-pVTZ//SMD for S_4-Cu^{II} complexes.

Structure	CSD code	X	S_1-Cu	S_2-Cu	S_3-Cu	S_4-Cu	S_1-Cu-S_3	
X-ray	VIMDOK[5]	2x$(ClO_4)^-$	2.292(1)		2.312(1)		179.97	
	THCDCU[6]	2x$(ClO_4)^-$	2.297(1)		2.308(1)		179.97	
	AVERAGE		2.288		2.309		179.97	
	STD		0.009		0.001		0.00	
SVWN			2.22404	2.19329	2.22647	2.20347	138.003	139.249
BP86			2.29967	2.27449	2.26201	2.29482	139.998	139.247
B3LYP			2.34365	2.31742	2.30139	2.3385	133.876	150.307
CAMB3LYP			2.29842	2.27664	2.29584	2.26126	141.493	141.228
B3PW91			2.29629	2.27368	2.29341	2.25962	141.329	140.979
M06L			2.30651	2.28226	2.30968	2.26995	140.615	140.435
M06			2.30137	2.27964	2.30062	2.26456	139.33	138.676
M062X			2.36365	2.33022	2.36167	2.3487	142.757	141.296
M06HF			2.37448	2.3507	2.37989	2.37222	142.803	140.695
B97D			2.32623	2.28273	2.33126	2.29836	138.728	137.243
ωB97xD			2.29945	2.27635	2.9655	2.26217	141.291	140.344
PBE0			2.29253	2.25795	2.28987	2.26893	141.645	142.794
TPSSTPSS			2.29588	2.26214	2.27138	2.29429	140.331	141.694
		AVERAGE	2.309398	2.281332	2.301542	2.28745	140.1692	141.0913
		STD	0.036106	0.036489	0.038303	0.043047	2.278663	2.998071

Table S36: Comparison of critical bonds (distances in angstroms) and angles (in degrees) of X-ray structures and structures calculated using DFT//cc-pVTZ//SMD for S_4-Cu^I complexes.

Structure	CSD code	X	S_1-Cu	S_2-Cu	S_3-Cu	S_4-Cu	S_1-Cu-S_3	S_2-Cu-S_4
SVWN			2.27351	2.22242	2.26467	2.22261	125.621	130.049
BP86			2.352	2.29595	2.34493	2.29705	127.553	131.496
B3LYP			2.39062	2.33982	2.40082	2.33559	129.67	133.292
CAMB3LYP			2.3751	2.36602	2.31355	2.31947	129.546	133.236
B3PW91			2.35553	2.36224	2.30937	2.30421	132.811	129.357
M06L			2.39071	2.31049	2.37209	2.31097	131.75	128.551
M06			2.37271	2.3241	2.38979	2.32168	131.939	128.433
M062X			2.46443	2.41359	2.46841	2.53599	150.741	116.362
M06HF			2.43758	2.48319	2.49014	2.53629	156.439	113.481
B97D			2.38125	2.31765	2.39834	2.32066	130.484	124.769
ωB97xD			2.36223	2.31529	2.38558	2.31208	132.854	128.13
PBE0			2.35783	2.29977	2.35039	2.30546	132.89	129.273
TPSSTPSS			2.34429	2.27173	2.33893	2.29358	131.178	127.842
		AVERAGE	2.373676	2.332482	2.371308	2.339665	134.1135	127.3285
		STD	0.043884	0.062409	0.059758	0.087691	8.627131	5.692079

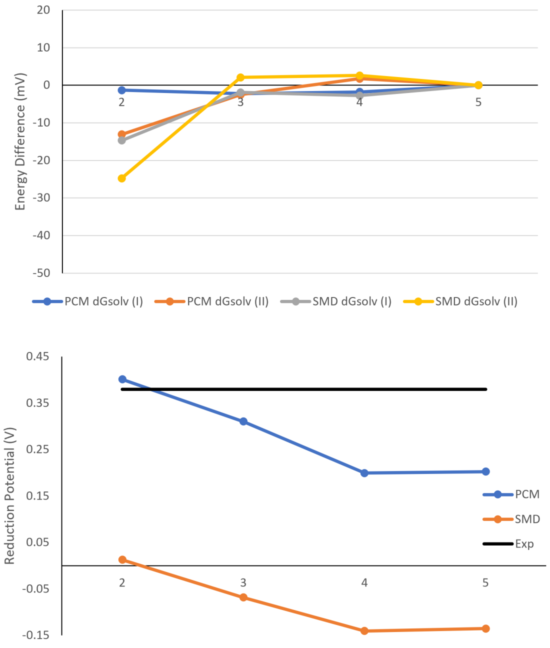

Figure S2: Convergence of EA, ΔG_{solv}(I) and ΔG_{solv}(II) (top); and calculated reduction potentials (bottom) with cc-pVnZ basis set for N_1S_3 complex.

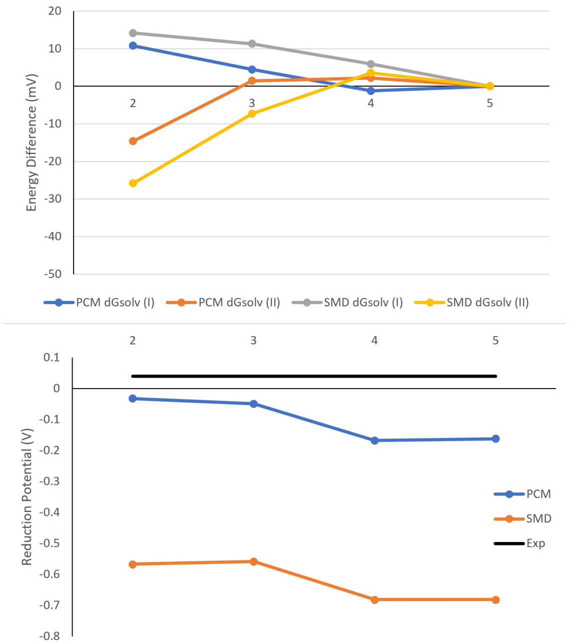

Figure S3: Convergence of EA, ΔG_{solv}(I) and ΔG_{solv}(II) (top); and calculated reduction potentials (bottom) with cc-pVnZ basis set for N_2S_2 complex.

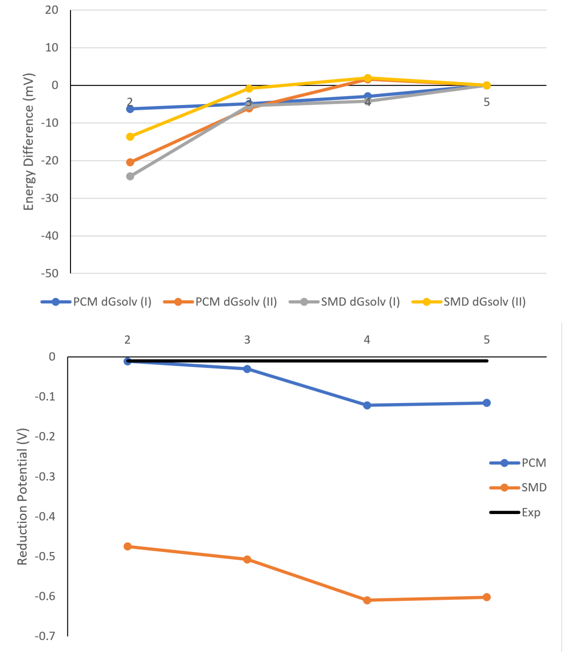

Figure S4: Convergence of EA, ΔG_{solv}(I) and ΔG_{solv}(II) (top); and calculated reduction potentials (bottom) with cc-pVnZ basis set for NSSN complex.

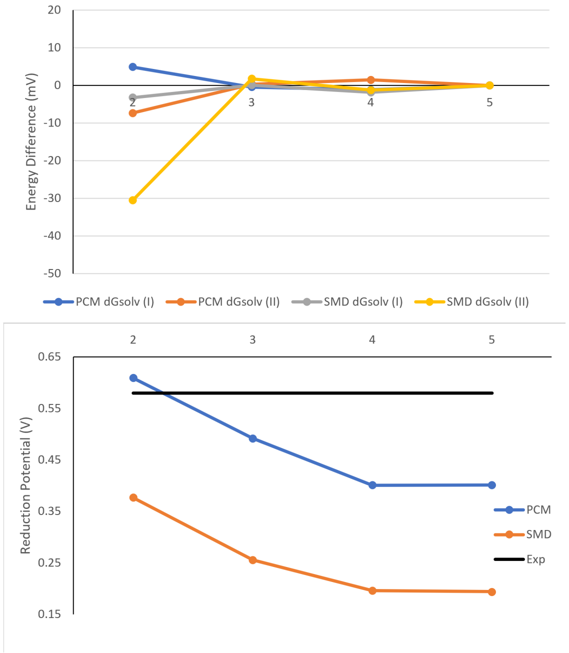

Figure S5: Convergence of EA, ΔG_{solv}(I) and ΔG_{solv}(II) (top); and calculated reduction potentials (bottom) with cc-pVnZ basis set for S_4 complex.

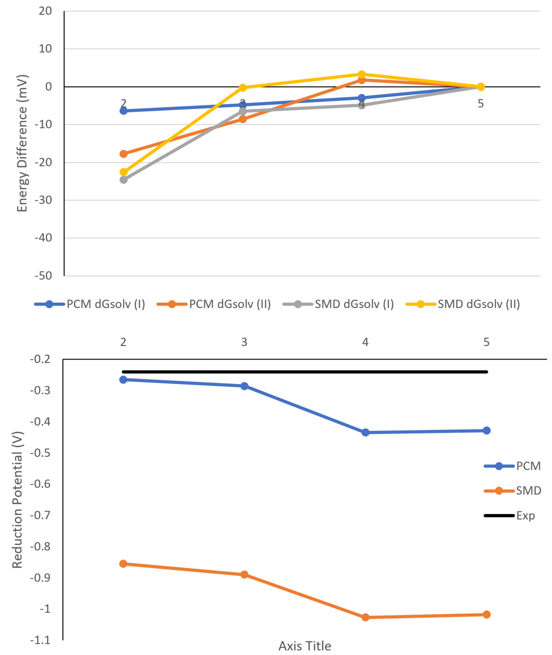

Figure S6: Convergence of EA, ΔG_{solv}(I) and ΔG_{solv}(II) (top); and calculated reduction potentials (bottom) with cc-pVnZ basis set for N_3S_1 complex.
